# Modulating the Electromechanical Response of Bio-Inspired Amino Acid-Based Architectures through Supramolecular Co-Assembly

**DOI:** 10.1021/jacs.2c06321

**Published:** 2022-09-27

**Authors:** Wei Ji, Bin Xue, Yuanyuan Yin, Sarah Guerin, Yuehui Wang, Lei Zhang, Yuanqi Cheng, Linda J. W. Shimon, Yu Chen, Damien Thompson, Rusen Yang, Yi Cao, Wei Wang, Kaiyong Cai, Ehud Gazit

**Affiliations:** † Key Laboratory of Biorheological Science and Technology, Ministry of Education, College of Bioengineering, Chongqing University, Chongqing 400044, P. R. China; ‡ The Shmunis School of Biomedicine and Cancer Research, George S. Wise Faculty of Life Sciences, Tel Aviv University, Tel Aviv 6997801, Israel; § National Laboratory of Solid State Microstructure, Department of Physics, Nanjing University, Nanjing, Jiangsu 210093, China; ∥ Chongqing Key Laboratory of Oral Diseases and Biomedical Sciences, Chongqing Municipal Key Laboratory of Oral Biomedical Engineering of Higher Education, Stomatological Hospital of Chongqing Medical University, Chongqing 401147, China; ⊥ Department of Physics, Bernal Institute, University of Limerick, Limerick V94 T9PX, Ireland; # CAEP Software Center for High Performance Numerical Simulation, Beijing 100088, China; ∇ Department of Chemical Research Support, Weizmann Institute of Science, Rehovot 76100, Israel; ○ School of Advanced Materials and Nanotechnology, Xidian University, Xi’an 710126, China

## Abstract

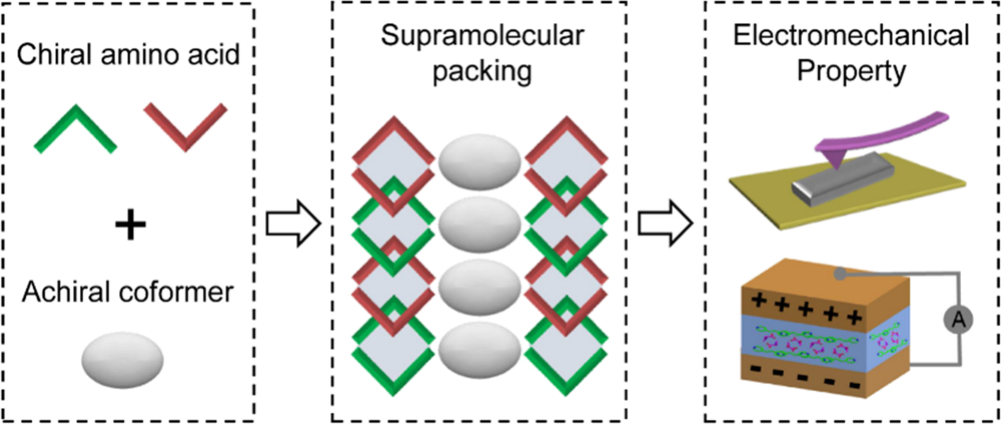

Supramolecular packing dictates the physical properties of bio-inspired molecular assemblies in the solid state. Yet, modulating the stacking modes of bio-inspired supramolecular assemblies remains a challenge and the structure–property relationship is still not fully understood, which hampers the rational design of molecular structures to fabricate materials with desired properties. Herein, we present a co-assembly strategy to modulate the supramolecular packing of N-terminally capped alanine-based assemblies (Ac-Ala) by changing the amino acid chirality and mixing with a nonchiral bipyridine derivative (BPA). The co-assembly induced distinct solid-state stacking modes determined by X-ray crystallography, resulting in significantly enhanced electromechanical properties of the assembly architectures. The highest rigidity was observed after the co-assembly of racemic Ac-Ala with a bipyridine coformer (BPA/Ac-DL-Ala), which exhibited a measured Young’s modulus of 38.8 GPa. Notably, BPA crystallizes in a centrosymmetric space group, a condition that is broken when co-crystallized with Ac-L-Ala and Ac-D-Ala to induce a piezoelectric response. Enantiopure co-assemblies of BPA/Ac-D-Ala and BPA/Ac-L-Ala showed density functional theory-predicted piezoelectric responses that are remarkably higher than the other assemblies due to the increased polarization of their supramolecular packing. This is the first report of a centrosymmetric-crystallizing coformer which increases the single-crystal piezoelectric response of an electrically active bio-inspired molecular assembly. The design rules that emerge from this investigation of chemically complex co-assemblies can facilitate the molecular design of high-performance functional materials comprised of bio-inspired building blocks.

## Introduction

Inspired by living systems, hierarchical supramolecular self-assembly produced by the bottom-up organization has received increasing interest for the design and fabrication of advanced functional materials.^1−10^ The long-range ordered spatial arrangement is formed under thermodynamic equilibrium conditions by the spontaneous aggregation of biomolecules (e.g., peptides and proteins) through noncovalent interactions, such as hydrogen bonding, π–π stacking, metal ion coordination, hydrophobic effects, etc.^11−21^ To extend this strategy for minimalistic building blocks with benefits of easy preparation, bio-degradability, and low cost, recent studies have shown that very simple amino acid and dipeptide molecules can also self-assemble and generate highly ordered structures to produce functional materials with unique physical properties.^22−31^ For example, piezoelectric coefficients of biological materials are generally low, usually in the range of 0.1–10 pm V^–1^, limiting their technological applications. As a single amino acid, glycine can self-assemble into β-type needle crystals exhibiting a remarkable high shear piezoelectric constant of 178 pm V^–1^ due to the high polarization of the molecular packing, which is comparable to inorganic materials such as barium titanate and lead zirconate titanate.^32^ Furthermore, glycine crystals were utilized to fabricate a piezoelectric polymer-based thin film for in vivo real-time sensing, actuation, and electricity generation.^33,34^ Tyrosine crystals with tightly packed dimer structures possess high rigidity and could be applied as functional elements in photo-waveguiding, mechano-responsive bending composites, and piezoelectric nanogenerators.^35^

The physical properties of molecular assembled materials are dictated by their supramolecular packing modes in the solid state.^36^ Understanding and controlling solid-state molecular arrangements are fundamental issues in encoding the desired physical properties of supramolecular materials. However, modulating the electromechanical properties of bio-inspired molecular assemblies remains a challenge. Inspired by previous studies, amino acid chirality plays a key role in controlling the geometry of the molecules and the handedness of the supramolecular organization,^37,38^ and co-assembly, the tactic employed by natural systems to expand the conformational space of supramolecular architectures, provides an efficient way to create highly functional and complex structures via noncovalent intermolecular interactions.^21,39,40^ Therefore, the molecular packing modes of bio-inspired amino acid-based architectures could be altered through the change in molecular chirality and co-assembly with additives. Single-crystal X-ray diffraction is an ideal methodology to provide atomically resolved structures and precisely establish the structure–property correlation.^41,42^ However, polymorphism is usually difficult to avoid during the assembly process, and thus, obtaining crystal structures remains a challenge.^43^ As a result, the relationship between molecular arrangement and properties of supramolecular assemblies is still not fully understood.

**Figure 1:**
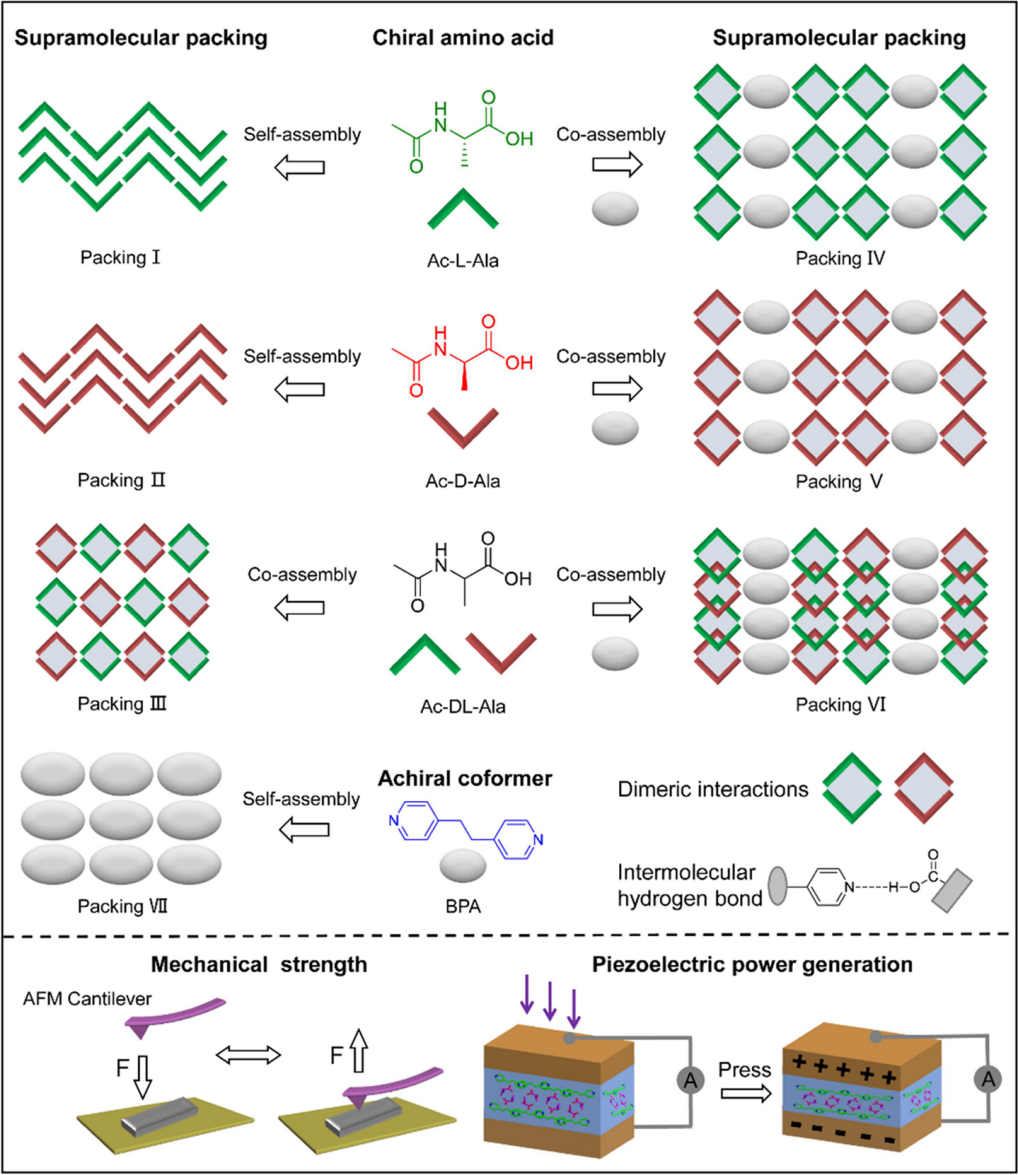
Schematic presentation of modulating the supramolecular packing of Ac-Ala molecules through racemic mixing and co-assembly with the BPA coformer, resulting in tunable electromechanical properties of supramolecular assemblies, including mechanical strength and piezoelectricity.

Herein, we present a supramolecular co-assembly approach to modulate the mechanical and piezoelectric properties of chiral acetylated alanine (Ac-Ala)-based assemblies with a nonchiral bipyridine coformer (1,2-bis(4-pyridyl)ethane, BPA) (Figure 1). The distinct solid-state stacking modes of supramolecular assemblies were determined by single-crystal X-ray diffraction, which resulted in significantly different electromechanical response of the Ac-Ala-based assemblies. Atomic force microscopy (AFM) nanoindentation experiments revealed that the mechanical properties of the crystals could be improved by racemic mixing and co-assembly with additives, with the highest value of Young’s modulus (38.8 GPa) and point stiffness (443.3 N/m) observed for the BPA/Ac-DL-Ala sample. Density functional theory (DFT) predictions combined with energy harvesting experiments showed that enantiopure co-crystals of BPA/Ac-D-Ala and BPA/Ac-L-Ala possessed remarkably higher piezoelectric response than the other crystals due to lower symmetry of the supramolecular packing. These results demonstrate the modulation of molecular packing and physical properties of supramolecular assemblies, which promotes the precise understanding of structure–property relationships. This work systematically builds on our previous studies using bipyridine derivatives as coformers to produce molecular-level design rules for co-crystal engineering.^44^ Compared to the noncentrosymmetric crystallizing BPE and BPY coformers used in previous studies, BPA molecules crystallize in the centrosymmetric structure without a piezoelectric response in the space group of *P*2_1_/*c*. Knowledge of the modulation of a centrosymmetric-crystallizing coformer into crystals through co-assembly with amino acid derivatives is still lacking.^45^ Unraveling this phenomenon could promote the understanding of the complexity and functionality of materials from multiple building blocks. Moreover, the majority of the literature on co-crystal engineering focuses on optimizing pharmaceutical properties such as bioavailability and solubility.^46^ As we are only beginning to understand how co-crystallization affects electromechanical properties, it is important to be systematic in our progression to identify real molecular effects as opposed to artifacts due to arbitrary differences in experimental conditions.

## Results and Discussion

**Figure 2:**
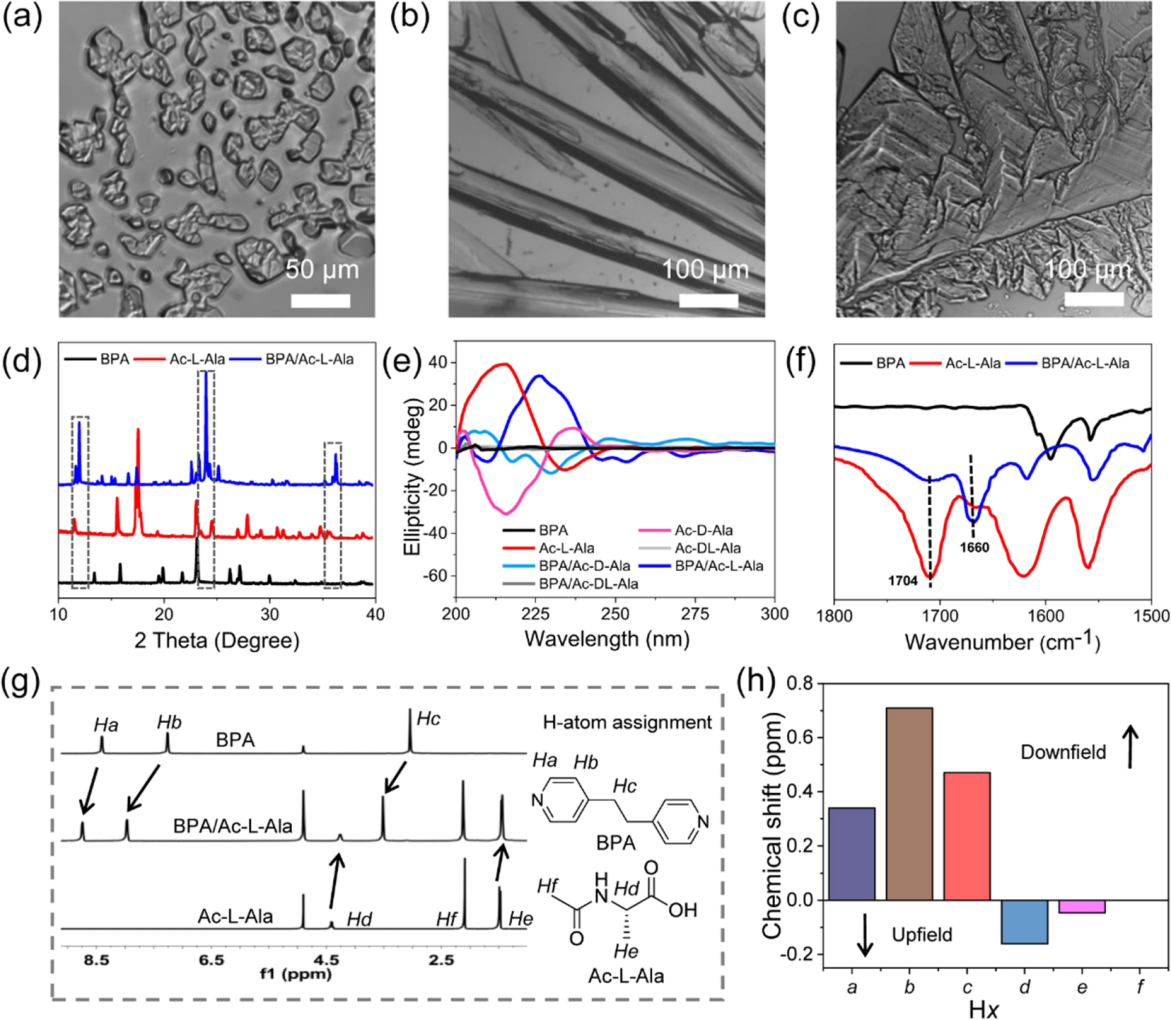
(a–c) Microscopy images of (a) BPA, (b) BPA/Ac-D-Ala, and (c) BPA/Ac-DL-Ala assemblies. (d) PXRD of BPA, Ac-L-Ala, and BPA/Ac-L-Ala assemblies. (e) CD spectra of the full set of alanine-based self- and co-assemblies. (f) FTIR spectra of BPA, Ac-L-Ala, and BPA/Ac-L-Ala self- and co-assemblies. (g) ^1^H NMR spectra of BPA, BPA/Ac-L-Ala, and Ac-L-Ala. (h) Chemical shift of ^1^H NMR of BPA/Ac-L-Ala, compared to the single components.

We chose Ac-Ala, the simplest nonaromatic chiral acetylated amino acid, and the BPA aromatic achiral coformer for the synthesis of co-assemblies due to the strong intermolecular hydrogen bonding between the carboxylic acid group and the pyridine (Figure 1). All crystalline solid samples were obtained by dissolving the powder of Ac-Ala and BPA in methanol and subsequent slow evaporation at ambient temperature. The molar ratio of BPA/Ac-Ala in the co-assemblies was 1:2, which reflected the number of pyridine and carboxylic acid groups in BPA and Ac-Ala, respectively. We subjected the systems to optical microscopy analysis and visualized the assembled morphology formed by pure Ac-Ala and the mixed systems with BPA. A fractal shape was observed for Ac-DL-Ala, while a plate-like morphology was detected in pure Ac-D-Ala and Ac-L-Ala assemblies. The difference in morphology indicated that co-assembly indeed occurred in the racemic Ac-DL-Ala mixture (Figure S1a–c). Pure BPA could self-assemble and form irregular block-shaped crystals. Needle-shaped assemblies were observed for BPA/Ac-D-Ala and BPA/Ac-L-Ala, while a racemic mixture of BPA/Ac-DL-Ala formed a dendrite-like structure, indicating the co-assembly of BPA and chiral Ac-Ala molecules (Figures 2a–c and S1d).

To gain more information of the molecular organizations, all samples were examined by powder X-ray diffraction (PXRD). The PXRD patterns for BPA, Ac-L-Ala, and BPA/Ac-L-Ala are shown in Figure 2d and Table S1. Pure BPA exhibited characteristic crystalline peaks at 2θ values of 13.40, 15.83, 19.88, 21.71, 23.10, 26.23, 27.15, and 29.94°, whereas Ac-L-Ala exhibited crystalline peaks at 11.52, 15.55, 17.54, 23.04, 24.50, and 27.87°. However, the PXRD pattern of the BPA/Ac-L-Ala mixture was distinguishable from the single-component assemblies and exhibited new peaks at 14.13, 15.03, 22.57, 23.96, 25.14, and 36.14°. Similarly, compared to their single components, new peaks in the diffraction patterns were also observed for BPA/Ac-D-Ala (11.96, 14.19, 25.10, and 36.20°) and BPA/Ac-DL-Ala (12.36° and 24.72°) (Figure S2a,b). The observable differences in the diffraction peaks indicated the co-assembly between BPA and Ac-Ala forming a new crystalline phase. Furthermore, compared to enantiopure assemblies, the appearance of new peaks and the absence of several original peaks were observed in the racemic mixtures, indicating that different molecular organizations were formed after racemic mixing (Figure S2c,d). Circular dichroism (CD) spectroscopy was used to study the supramolecular chirality of the different samples (Figure 2e). No obvious chirality signals were detected for the samples of BPA, Ac-DL-Ala, and BPA/Ac-DL-Ala. The pure Ac-L-Ala assemblies exhibited positive and negative Cotton effects at 216 and 234 nm, respectively, with the mirrored signal observed in Ac-D-Ala assemblies with Cotton effects at 237 and 216 nm. Completely different Cotton effects were detected in the BPA/Ac-L-Ala (227, 254, and 210 nm) and BPA/Ac-D-Ala (209, 218, and 230 nm) mixtures, reflecting the formation of new molecular arrangements after co-assembly. The thermal stability of the self- and co-assemblies was investigated using thermogravimetric analysis (Figure S3). The stable weight loss started at 123.6, 163.2, 164.8, and 173.4 °C for BPA, Ac-L-Ala, Ac-D-Ala, and Ac-DL-Ala, respectively. Compared to single components, higher thermal stability was observed for the BPA/Ac-L-Ala (174.8 °C), BPA/Ac-D-Ala (167.2 °C), and BPA/Ac-DL-Ala (190.6 °C) co-assemblies, suggesting the stable molecular packing in the assemblies.

Fourier-transform infrared (FTIR) spectroscopy was employed to understand the intermolecular hydrogen bonding between the carboxylic acid group and the pyridine. As shown in Figure 2f, the absorption peak located at 1704 cm^–1^ was assigned to the C=O stretching vibration of the carboxylic acid in Ac-L-Ala, which was significantly damped and shifted to a lower wavenumber of 1660 cm^–1^ after co-assembly with BPA, indicating the formation of intermolecular hydrogen bonding between the carboxylic acid group and the pyridine. The peak was assigned to N–H stretching vibration of the amide in Ac-L-Ala (3330 cm^–1^), which was also shifted to a lower wavenumber (3298 cm^–1^) in the mixture of BPA/Ac-L-Ala, marking a corresponding change of the amide interactions after co-assembly (Figure S4a). Similar shifting of FTIR peaks for the carboxylic acid and amide bands was observed for BPA/Ac-D-Ala and BPA/Ac-DL-Ala (Figure S4b–e). ^1^H NMR experiments were also performed to examine the chemical shifts of hydrogen atoms after co-assembly. The H-atom assignments of BPA (*Ha*, *Hb*, *Hc*) and Ac-Ala (*Hd*, *He*, *Hf*) are shown in Figures 2g and S5. In the BPA/Ac-L-Ala mixture, the hydrogen atoms of BPA (*Ha*, *Hb*, *Hc*) underwent a pronounced downfield shift, while a visible upfield shift was observed for the hydrogen atoms of Ac-L-Ala (*Hd*, *Hf*), indicating the formation of hydrogen bonding between the carboxylic acid and pyridine (Figure 2h). Compared with their single components, similar chemical shifts were observed for the co-assembly of BPA/Ac-D-Ala and BPA/Ac-DL-Ala (Figure S5). These results suggested that the intermolecular hydrogen bonding between the carboxylic acid and pyridine was the main enthalpic driving force for co-assembly.

**Figure 3:**
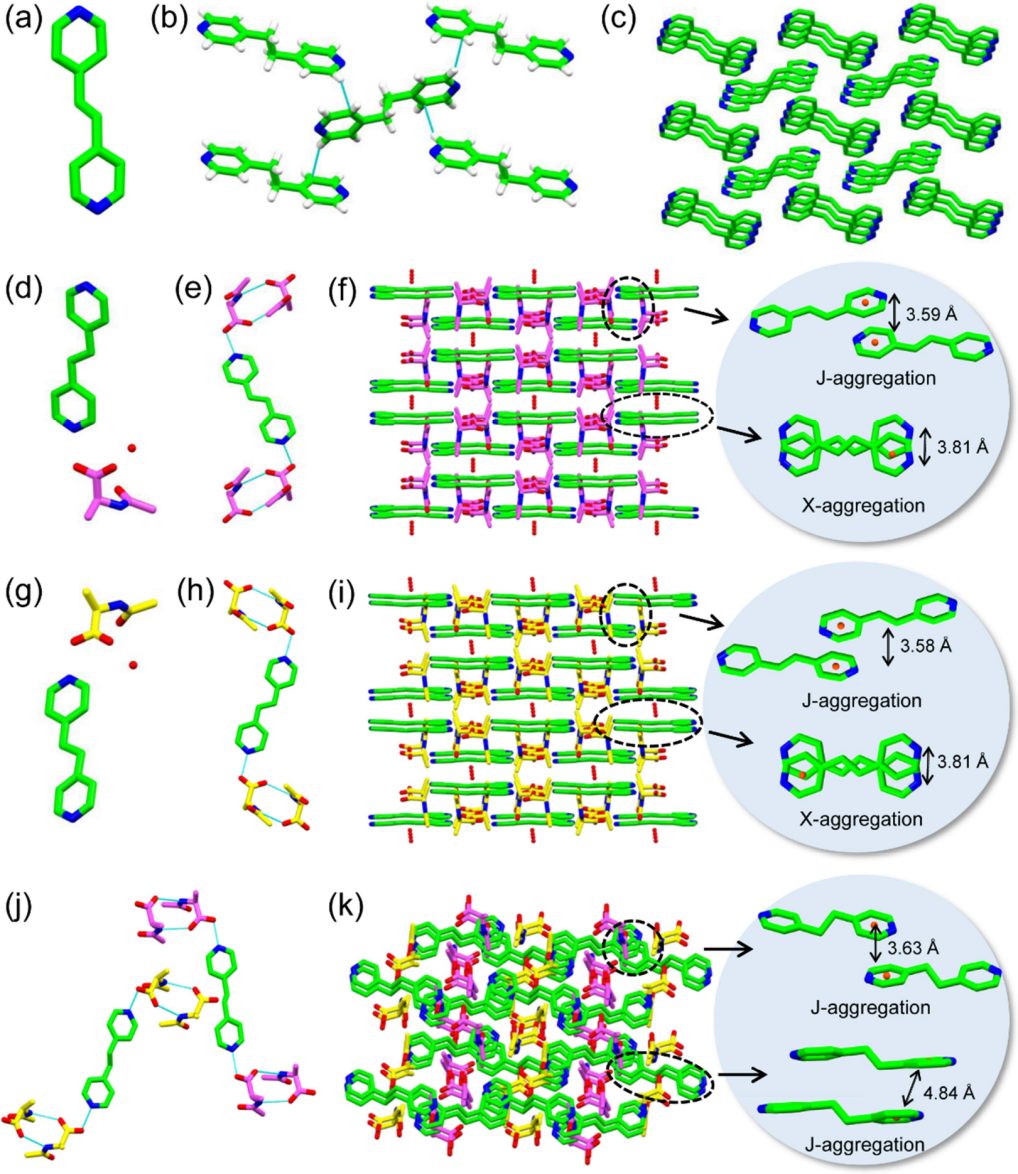
(a–c) Single-crystal structure of BPA: (a) asymmetric unit, (b) intermolecular CH-π hydrogen bonding between BPA molecules, (c) higher-order molecular packing in the crystallographic *c* direction. (d–f) Co-crystal structure of BPA/Ac-L-Ala: (d) asymmetric unit, (e) intermolecular hydrogen bonding between BPA and the Ac-L-Ala dimer, and (f) higher-order hybrid molecular packing with characteristic aromatic interactions between adjacent BPA molecules. (g–i) Co-crystal structure of BPA/Ac-D-Ala: (g) asymmetric unit, (h) intermolecular hydrogen bonding between BPA and the Ac-D-Ala dimer, and (i) higher-order hybrid molecular packing with characteristic aromatic interactions between adjacent BPA molecules. (j,k) Co-crystal structure of BPA/Ac-DL-Ala: (j) intermolecular hydrogen bonding between BPA and Ac-L-Ala or Ac-D-Ala dimers and (k) higher-order hybrid molecular packing with characteristic aromatic interactions between adjacent BPA molecules. (f, i, k) Modulated aromatic interactions between the BPA molecules present in the structure have been highlighted, and enlarged images are shown on the right. The carbon atoms of BPA, Ac-L-Ala, and Ac-D-Ala are colored in green, pink, and yellow, respectively. Heteroatom nitrogen and oxygen are colored in blue and red, respectively.

To gain insight into the supramolecular arrangements of Ac-Ala-based assemblies at the atomic level, the crystal structures of the diffraction quality samples were solved and analyzed in detail (Table S2). The packing mode of BPA crystals in this study was found to be the same as the previously reported BPA crystal structure.^47^ One molecule of BPA crystalized in the asymmetric unit without any solvent molecules in monoclinic space group *P*2_1_/*c* (Figure 3a). Each BPA molecule is connected with four adjacent molecules through intermolecular CH-π hydrogen bonding (C5–H5···C2’ and C5’–H5’···C2) with a C···H distance of 2.794 Å (Figure 3b). The higher-order packing pattern of the BPA crystal is shown in Figure 3c, where no significant π–π stacking between aromatic rings is detected in the structures due to the long centroid-to-centroid distance (8.159 Å) (Figure S6). Ac-L-Ala and Ac-D-Ala produced mirrored unit cell and higher-order packing in their crystal structures (Figure S7a–f). Ac-L-Ala and Ac-D-Ala crystallized in asymmetric units without any solvent molecules in the orthorhombic *P*2_1_2_1_2_1_ space group with one molecule per asymmetric unit (Figure S7a,d). The intermolecular hydrogen bonds between Ac-L-Ala and Ac-D-Ala molecules and four adjacent molecules were mediated through both carboxylic acid and amide groups. The head-to-tail H-bonded connection of the molecules produced a zigzag molecular chain in the crystallographic *a*-direction (Figure S7b,e). Two of the hydrogen bonds were formed via the carboxylic acid group (O3···H1–N1 and O2–H2A···O1) and two hydrogen bonds were mediated via the amide group (N1–H1···O3 and O1···H2A–O2), with a O3···H1 distance of 2.136 Å and H2A···O1 distance of 1.728 Å. The higher order packing of the Ac-L-Ala and Ac-D-Ala crystals along the crystallographic *c* direction is shown in Figure S7c,f.^48^ However, the structure of Ac-DL-Ala racemate was significantly different from the pure Ac-L-Ala and Ac-D-Ala. Racemic Ac-DL-Ala crystalized in the asymmetric unit with four molecules in the monoclinic space group *P*2_1_/*c* (Figure S7g). Interestingly, face-to-face dimeric structures were formed by two Ac-L-Ala or two Ac-D-Ala molecules via two intermolecular hydrogen bonds (Figure S7h). The D- and L-type dimers were further connected through an O–H···O hydrogen bond, producing 2D sheet-like structures. Compared with pure Ac-L-Ala and Ac-D-Ala, a denser high-order packing was obtained in the racemic Ac-DL-Ala crystal structure (Figure S7i).

Crystallization of BPA and Ac-L-Ala together resulted in the formation of a co-crystal containing one molecule of planar BPA, one molecule of Ac-L-Ala, and one molecule of water in the asymmetric unit with an orthorhombic space group *C*222, producing a dramatically different molecular arrangement compared to the individual single-crystal structures (Figure 3d).^44^ Notably, Ac-L-Ala formed a strong dimer structure stabilized by two intermolecular hydrogen bonds, which was further connected with a BPA molecule through intermolecular hydrogen bonding between carboxylic acid and pyridine groups (Figure 3e). Therefore, the crystal structure results were in good agreement with the FTIR and ^1^H NMR experiments. When viewed along the *c* axis, the higher-order packing of the co-crystal showed a hybrid arrangement composed of BPA molecular stacking and the Ac-L-Ala dimers (Figure 3f). In this packing mode, the interactions between adjacent BPA molecules were remarkably different from those observed in the BPA single-crystal structure, indicating the change of BPA interactions in the co-crystal structure of BPA/Ac-L-Ala. Two types of π–π aromatic interactions were detected, namely, head-to-tail configuration (J-aggregation) and cross-stacking mode (X-aggregation), with a centroid-to-centroid stacking distance of 3.59 and 3.81 Å between overlapping pyridine rings, respectively. The co-crystal of BPA/Ac-D-Ala produced very similar unit cell parameters and mirrored higher-order packing geometries compared to BPA/Ac-L-Ala in an orthorhombic space group *C*222. Thus, the asymmetric unit, molecular interactions, and high-order crystal packing in the BPA/Ac-D-Ala co-crystal were very similar to that of BPA/Ac-L-Ala, with near-identical aromatic J- and X-aggregation stacking patterns (centroid-to-centroid distance of 3.58 and 3.81 Å, respectively; Figure 3g–i). By contrast, the co-crystal of BPA/Ac-DL-Ala showed a completely different supramolecular packing. Racemic BPA/Ac-DL-Ala crystalized in the asymmetric unit with a triclinic space group *P*-1, containing two BPA molecules, one Ac-L-Ala molecule, and three Ac-D-Ala molecules (Figure S8a). No solvent molecules were found in the asymmetric unit. Ac-L-Ala and Ac-D-Ala separately interacted with BPA to produce two different molecular chains comprised of the D- and L-type dimeric interactions and intermolecular hydrogen bonding between carboxylic acid and pyridine groups (Figure 3j). Notably, one was composed of BPA/Ac-L-Ala and the other one of BPA/Ac-D-Ala. Each individual isomer formed a layer-by-layer arrangement with BPA in the higher-order packing, producing a highly dense and complex 3D packing network (Figure 3k). Three types of J-aggregation interactions were observed between pyridine rings, displaying centroid-to-centroid distances of 3.63, 4.84, and 3.61 Å (Figures 3k and S8b). These results suggested that the co-crystallization and racemic mix could improve the combined effect of noncovalent interactions (e.g., hydrogen bonding and π–π stacking), producing strong supramolecular packing networks.

**Figure 4:**
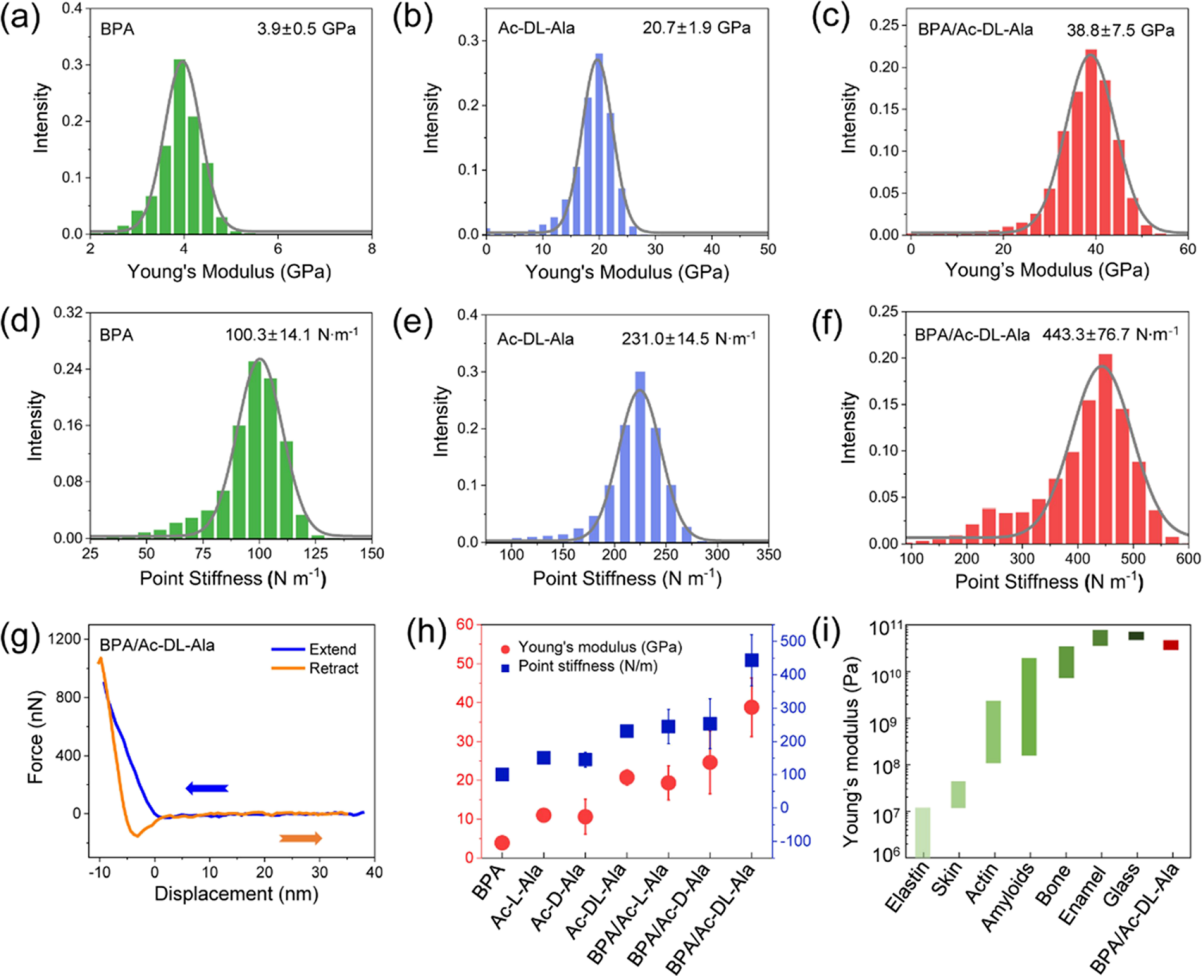
(a–c) Statistical Young’s modulus distributions of (a) BPA, (b) Ac-DL-Ala, and (c) BPA/Ac-DL-Ala. (d–f) Statistical point stiffness distributions of (d) BPA, (e) Ac-DL-Ala, and (f) BPA/Ac-DL-Ala. (g) Typical force–displacement traces of BPA/Ac-DL-Ala; the left arrow represents expansion, and the right arrow represents retraction. (h) Statistical Young’s modulus and point stiffness values for all the crystals. (i) Comparison of the Young’s modulus of different biological and nonbiological materials,^49^ such as elastin, skin, actin, amyloids, bone, enamel, and glass.

Exploiting the mechanical properties of supramolecular structures is important for a plethora of materials applications spanning tissue engineering, polymer science, pharmaceuticals, therapeutics, and optoelectronic devices. AFM nanoindentation was applied to investigate the influence of supramolecular packing on the mechanical properties of the full set of alanine-based crystals. The AFM cantilever tip was contacted on the surface of the crystal sample and then retracted, with the load/unload speed kept at 2 μm s^–1^ (Figure S9). The force–distance curve was obtained using the quantitative imaging model of the probe, which represented the function of the force applied to the tip and the indentation depth (Figures 4g and S10). The Young’s modulus and point stiffness could be calculated from the force–displacement traces using the Hertz model. As shown in Figures 4b,e and S11a–d, the statistical values of Young’s modulus along the thickness direction were 11.0 ± 1.5, 10.6 ± 4.5, and 20.7 ± 1.9 GPa for Ac-L-Ala, Ac-D-Ala, and Ac-DL-Ala, respectively. The corresponding point stiffness values of the crystals were 151.1 ± 10.5, 145.2 ± 22.8, and 231.0 ± 14.5 N m^–1^, respectively. Compared with pure Ac-L-Ala and Ac-D-Ala crystals, racemic Ac-DL-Ala exhibited higher Young’s modulus and point stiffness values with an order of Ac-DL-Ala > Ac- (D or L)-Ala. This was attributed to the tightly packed dimeric structures and dense crystal network of the racemic Ac-DL-Ala. For the BPA crystal, the statistical values of Young’s modulus and point stiffness were found to be 3.9 ± 0.5 GPa and 100.3 ± 14.1 N m^–1^, respectively (Figure 4a,d). After co-assembly with Ac-Ala, the BPA/Ac-L-Ala, BPA/Ac-D-Ala, and BPA/Ac-DL-Ala co-crystals exhibited Young’s modulus values of 19.3 ± 4.4, 24.6 ± 8.1, and 38.8 ± 7.5 GPa, respectively. The corresponding point stiffness values of the co-crystals were 244.8 ± 51.2, 253.2 ± 74.3, and 443.3 ± 76.7 N m^–1^, respectively (Figures 4c,f and S11e–h). The results indicated that the mechanical properties of the racemic alanine-based co-crystals were higher than those of the pure D- or L-form with a sequence of BPA/Ac-DL-Ala > BPA/Ac-(D or L)-Ala. Notably, co-assembly enhanced the mechanical properties compared to the corresponding single-component crystals, resulting in rigidity orders of [BPA/Ac-D-Ala > Ac-D-Ala, BPA], [BPA/Ac-L-Ala > Ac-L-Ala, BPA], and [BPA/Ac-DL-Ala > Ac-DL-Ala, BPA] (Figure 4h). This was ascribed to the tightly packed supramolecular organization of the co-crystals through π–π stacking and intermolecular hydrogen bonding. Compared to a broad range of biological and nonbiological materials (Figure 4i),^49^ the Young’s modulus value of the BPA/Ac-DL-Ala co-crystals (38.8 GPa) presented in this study is higher than those of biomaterials (e.g., elastin, skin, and actin) and is close to those of enamel and glass. A higher Young’s modulus implies a higher mechanical to electrical energy conversion efficiency. The electromechanical coupling factor , where *d*_*ij*_^2^ is the piezoelectric coefficient, *E*_Y_ is the Young’s modulus, and ε_d_ is the dielectric constant, is a parameter evaluating the conversion efficiency from mechanical to electrical energy.^50^ In the current study, the high Young’s modulus values of the noncentrosymmetric BPA/Ac-L-Ala and BPA/Ac-D-Ala crystals imply a strong piezoelectric response, which supports the feasibility of bio-inspired materials for the fabrication of bio-integrated microdevices that combine high structural stability, tailored optoelectronics, and significant energy harvesting properties.^10^

The macroscopic mechanical properties of materials are mainly controlled by the evolution of their microscopic electronic structure under strain. Therefore, we set out to investigate the intermolecular interactions of the seven molecular crystals studied, aiming to provide insight into each corresponding mechanical property. The analysis of intermolecular interactions was carried out with the CrystalExplorer package, including the Hirshfeld surface, fingerprint plot, and distribution of various atomic contacts.^51^

In the pristine BPA crystal, the hydrogen bonding was mainly composed of N···H and C···H contacts. In contrast, in the pristine Ac-L-Ala and Ac-D-Ala crystals, the hydrogen bonding was mainly composed of O···H contacts, as indicated by red dots on the Hirshfeld surfaces shown in Figures S12–14.

When assembling the BPA/Ac-L-Ala and BPA/Ac-D-Ala co-crystals, the intermolecular contacts surrounding the Ac-L-Ala and Ac-D-Ala molecules presented little variations, as shown in Figures S13 and S14. However, the interactions surrounding the BPA molecules showed significant change, which alter the mechanical properties of the co-crystals. In the BPA/Ac-L-Ala and BPA/Ac-D-Ala co-crystals, the proportion of weak N···H (2.77 Å) and C···H (3.84 Å) hydrogen bonding surrounding the BPA molecules decreased and the strong O···H (1.62 Å) hydrogen bonding was formed instead, as shown by the pronounced spike in their 2D fingerprint plots. The O···H contacts occupied ∼27% of the Hirshfeld surface of the BPA molecule in the co-crystals. Compared with the pristine BPA crystal, another significant change in the BPA/Ac-L-Ala and BPA/Ac-D-Ala co-crystals was the presence of aromatic π···π interactions (marked by C···C and N···N interatomic close contacts), which occupied ∼14% of the Hirshfeld surface of the BPA molecule in the co-crystals, as shown in Figure S12. Similar interactions were detected in the BPA/Ac-DL-Ala co-crystal. Still, the strong hydrogen bonding included O···H (2.00 Å) and N···H (1.69 Å) contacts, as indicated by the two pronounced spikes in their 2D fingerprint plots. In terms of π···π contacts, the proportion of the newly appeared aromatic interactions in the BPA/Ac-DL-Ala co-crystal was less than in the BPA/Ac-L-Ala and BPA/Ac-D-Ala co-crystals. Hydrogen bonding cooperativity and aromatic interactions have a buffering effect against mechanical stimuli,^52,53^ and the BPA/Ac-L-Ala, BPA/Ac-D-Ala, and BPA/Ac-DL-Ala co-crystals presented significantly smaller strain under a given stress, resulting in much higher Young’s modulus values compared to the pristine BPA, Ac-L-Ala, Ac-D-Ala, or Ac-DL-Ala crystals.

Comparing the Ac-DL-Ala co-crystal with the pristine Ac-L-Ala and Ac-D-Ala crystals, the *d*_e_ and *d*_i_ values in the two pronounced spikes of the 2D fingerprint plots decreased (Figures S13 and S14), indicating that the O···H hydrogen bonding became stronger, conferring the Ac-DL-Ala co-crystal with more robust capability of suppressing strain under stress. For the BPA/Ac-DL-Ala, BPA/Ac-L-Ala, and BPA/Ac-D-Ala co-crystals, it is interesting to notice two changes in their intermolecular interactions, as shown in Figures S13 and S14. The first change is the translation of the strong O···H (1.73 Å) into even stronger N···H (1.69 Å) in the Ac-DL-Ala co-crystal, and the second is the presence of a considerable amount of weak C···H (3.86 Å) hydrogen bonding. Such an electronic structure variation confers the BPA/Ac-DL-Ala co-crystal with a stronger capability of suppressing variation under strain, thereby showing higher Young’s modulus than the BPA/Ac-L-Ala and BPA/Ac-D-Ala co-crystals.

As Ac-L-Ala and Ac-D-Ala showed highly similar interatomic interactions in each pristine crystal or in the corresponding co-crystals formed with BPA, as shown in Figures S13 and S14, and Ac-L-Ala and Ac-D-Ala presented similar Young’s modulus, as did BPA/Ac-L-Ala and BPA/Ac-D-Ala.

On account of the different supramolecular polarization and stiffness, noncentrosymmetric crystals with diverse packing networks generate different piezoelectric responses. Thus, any crystal that belongs to a noncentrosymmetric space group is expected to demonstrate nonzero piezoelectric response through linear coupling of electrical and mechanical energy. Here, we used DFT calculations to predict the piezoelectric coefficients of the Ac-Ala-based series of crystals. The primary factor indicative of the piezoelectric response, *d_ij_* measured in pC/N, is the ratio of the anisotropic piezoelectric polarization in C/m^2^ to the relevant elastic stiffness constant in GPa. Thus, a material with naturally high piezoelectricity is likely to have high polarization in its unit cell or low stiffness along a particular crystallographic plane.

**Figure 5:**
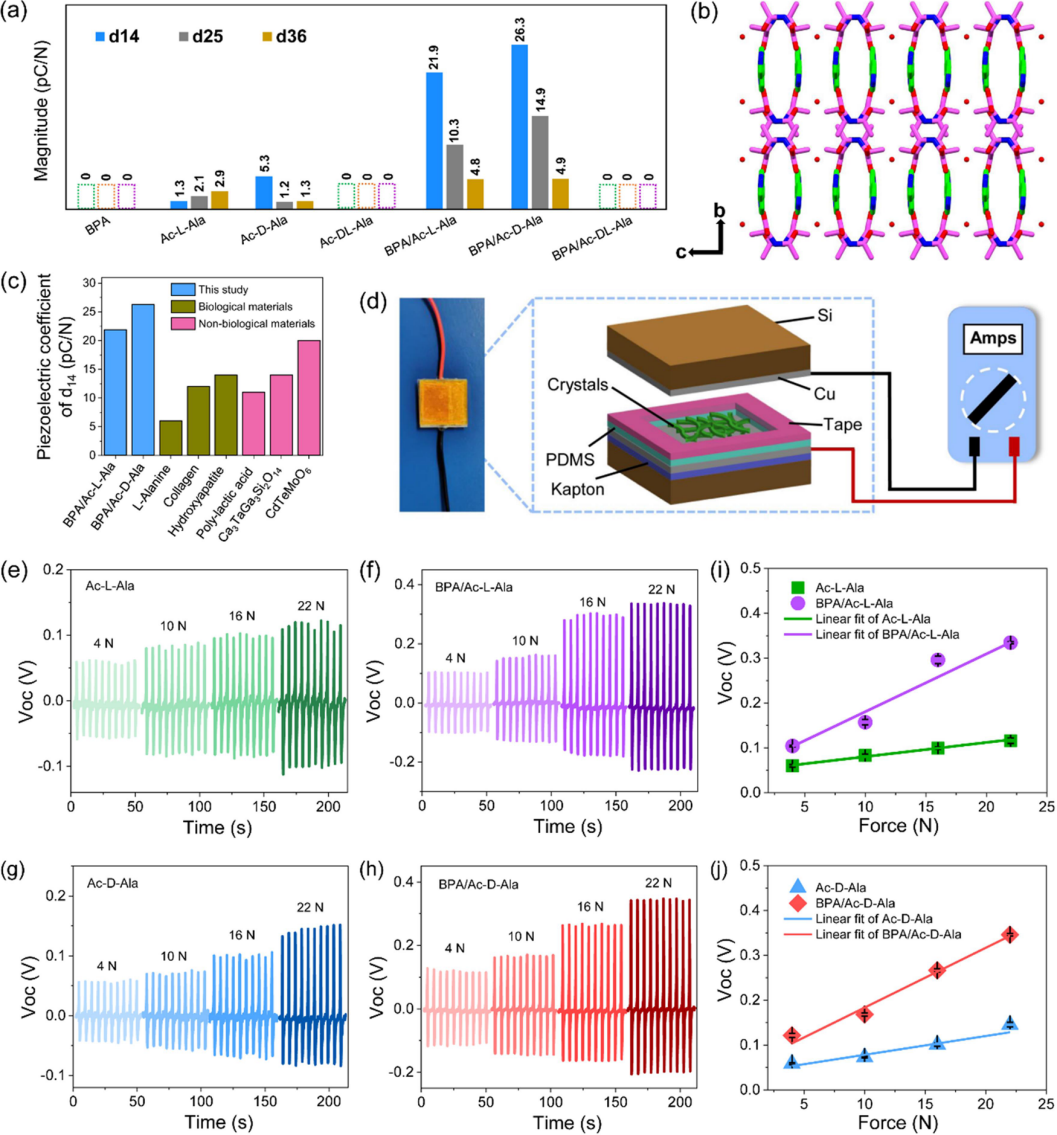
(a) Predicted absolute piezoelectric strain constants, *d_ij_*, of all the crystals. (b) Porous structure of BPA/Ac-D-Ala crystals. (c) Comparison of the piezoelectric coefficient *d*_14_ of selected different biological and nonbiological materials, as well as the crystals presented herein. The relevant references are shown in Table S8. (d) Schematic of the nanogenerator based on Ac-Ala crystals. The inset on the left shows a photograph of the coin-sized nanogenerators. (e–h) Open-circuit voltage of (e) Ac-L-Ala, (f) BPA/Ac-L-Ala, (g) Ac-D-Ala, and (h) BPA/Ac-D-Ala crystal-based generators obtained by applying forces of 4, 10, 16, and 22 N. (i,j) Linear fitting of the open-circuit voltage of (i) Ac-L-Ala, BPA/Ac-L-Ala and (j) Ac-D-Ala, BPA/Ac-DL-Ala crystal-based nanogenerators as a function of the applied force from 4 to 22 N.

DFT calculations were used to predict the piezoelectric coefficients of the Ac-Ala-based crystals. The optimized structures of the crystals used for prediction are shown in Figure S15. The predicted piezoelectric responses are summarized in Figure 5a and Tables S3–7. BPA, racemic Ac-DL-Ala, and BPA/Ac-DL-Ala crystallized in centrosymmetric space groups, which do not demonstrate piezoelectricity (all tensor values = 0). Both BPA/Ac-L-Ala and BPA/Ac-D-Ala crystallized in an orthorhombic space group, allowing three shear piezoelectric constants (indexed by *d*_14_, *d*_25_, and *d*_36_). Thus, while there is no net dipole in the equilibrium unit cell, the application of a shearing force to any axis will generate a surface charge along that axis. These two crystals are predicted to show near-identical piezoelectric properties. By contrast, for the two single crystals of Ac-L-Ala and Ac-D-Ala, the predicted maximal piezoelectric strain constants were *d*_36_ = 2.9 pC/N and *d*_14_ = 5.3 pC/N, respectively. Looking at the single-crystal charge tensors, the small *e*_25_ values of 0.01 C/m^2^ are among the lowest charge constants predicted for all the crystals, emphasizing the role of co-crystallization in increasing the available piezoelectric surface charge in molecular crystals. However, in the co-crystals, straining the *b* axis results in the higher *e*_25_ values of 0.08 C/m^2^ in BPA/Ac-D-Ala and 0.05 C/m^2^ in BPA/Ac-L-Ala. The higher polarization in the right-handed BPA/Ac-D-Ala crystal is due to a slight asymmetry in the BPA molecular packing in the porous co-crystal, leading to higher generated charge under an applied force. The co-crystals showed similar maximal piezoelectric strain constant *d*_max_ = *d*_14_ of 26.3 and 21.9 pC/N for BPA/Ac-D-Ala and BPA/Ac-L-Ala, respectively, as the axis of the highest charge does not correspond to the axis of the lowest stiffness. The porous structure of the BPA/Ac-L-Ala and BPA/Ac-D-Ala co-crystals produced high longitudinal stiffness in the direction of the continuous pore walls along the *a* axis, which promoted ionic displacement per unit force and resulted in higher piezoelectric response (Figures 5b and S16). The piezoelectric coefficient *d*_14_ values of BPA/Ac-L-Ala and BPA/Ac-D-Ala co-crystals were comparable to those of diverse materials (Figure 5c), including biological materials such as L-alanine (6 pC/N), collagen (12 pC/N), and hydroxyapatite (14 pC/N), and nonbiological materials such as poly-lactic acid (11 pC/N), Ca_3_TaGa_3_Si_2_O_14_ (14 pC/N), and CdTeMoO_6_ (20 pC/N). The relevant references are shown in Table S8. The results show that the maximum piezoelectric response of the BPA/Ac-D-Ala co-crystal is over twice those of collagen and poly-lactic acid materials and is comparable to that of CdTeMoO_6._ Furthermore, all four crystals showed significant piezoelectric voltage constants, indicating their potential for energy harvesting applications. The calculated *g*_max_ values were *g*_36_ = 109, *g*_14_ = 163, *g*_14_ = 477, and *g*_14_ = 545 mV m/N for Ac-L-Ala, Ac-D-Ala, BPA/Ac-L-Ala, and BPA/Ac-D-Ala, respectively. The values predicted for the co-crystals are higher than the shear, longitudinal, and transverse responses of lead zirconium titanate and match those for other high-performance crystals such as Bi_3_BO_6_.^54^

To further explore the piezoelectric power generation of the Ac-Ala-based crystals, coin-size nanogenerators were designed and fabricated by tightly sandwiching the Ac-Ala-based sample powder between two Al-coated silicon substrates that were connected to an external low-noise voltage preamplifier via copper wires (Figure 5d). Upon applying the pressing force to the molecular devices by a linear actuator, an electric dipole could be generated, resulting in an electrical current flowing to the top electrode. The current flowed back to the bottom electrode when the pressing force was released and the crystal film was no longer compressed. The resulting electrical output signal of open-circuit voltage (*V*_oc_) was collected by applying a pressing force ranging from 4 to 22 N (Figure 5e–h). Under an applied force of 4 N, the *V*_oc_ value reached 0.05 and 0.06 V for Ac-L-Ala- and Ac-D-Ala-based nanogenerators, while the *V*_oc_ values of 0.10 and 0.12 V were collected for BPA/Ac-L-Ala- and BPA/Ac-D-Ala-based nanogenerators, respectively. The output voltage was found to increase more significantly with the applied force. When the pressing force was increased to 22 N, the *V*_oc_ reached values of 0.12, 0.15, 0.32, and 0.35 V for the nanogenerators from Ac-L-Ala, Ac-D-Ala, BPA/Ac-L-Ala, and BPA/Ac-D-Ala, respectively. In particular, the applied force-dependent open-circuit voltages from 2 to 22 N showed a good linear fit, suggesting a stable piezoelectric response of the Ac-Ala-based crystals (Figure 5i,j). Notably, higher power generation was observed for the co-crystals of BPA/Ac-L-Ala and BPA/Ac-D-Ala compared to the enantiopure crystals of Ac-D-Ala and Ac-D-Ala, in good agreement with the DFT-predicted piezoelectric responses. In contrast, the racemic mixtures (Ac-DL-Ala and BPA/Ac-DL-Ala) generated centrosymmetric space groups that precluded piezoelectricity. We note that the measured voltages, while significant, do not quantitatively correspond to the predicted single-crystal voltage constants. Two reasons may account for the fact that the device measurements may not have achieved the predicted theoretical upper limit of performance. First, the energy harvesting device contains an active layer which can be considered as a polycrystalline film. Naturally grown biomolecular polycrystalline films always have a lower effective piezoelectric response compared to their single-crystal counterparts, since each anisotropic single-crystal response contributes to a net damped, nonmaximal film response in the chosen direction. Second, the energy harvesting device reaps longitudinal energy, whereas the predicted maximal piezoelectric response can only be induced via shearing. The measured voltages are generated by the effective longitudinal response of the material under uncomplicated device operating conditions. These results demonstrated that the fabricated nanogenerators based on the Ac-Ala crystals hold promising applications in energy harvesting with tunable piezoelectricity. These results revealed that achiral molecules could be co-crystallized with chiral molecules to significantly increase their electromechanical response. By disrupting the ordered symmetry of strongly hydrogen-bonded assemblies, we demonstrate in this work that we can systematically increase flexibility and polarization of candidate piezoelectric co-crystal formulations.

In the present work, then we added a new summary of how the current dataset adds to the knowledge on electromechanical properties, demonstrating the generality of our co-assembly approach to studying structure–function relations. Looking at our models across this study and our previous co-crystal work,^32,44,54,55^ clear trends are beginning to emerge regarding structural and chemical features that modulate the piezoelectric performance of bio-inspired co-crystals. (i) Co-crystallization can lower the symmetry of the system to maximize the number of nonzero piezoelectric constants, providing multiple actuation directions that can be exploited in devices. Changing symmetry can change the stiffness; for example, orthorhombic co-crystals tend to have high shear stiffness which reduces the piezoelectric strain constants, while the opposite is true for monoclinic co-crystals. (ii) Insertion of coformers can stiffen the assembly but can also change the polarity. For example, coformers can disrupt and resculpt long-range hydrogen bonding and π···π stacking networks, with aromatic or zwitterionic molecules tending to self-assemble as more polar single crystals and co-crystals. (iii) Solvents of crystallization are effective in reducing the shear stiffness and increasing the polarity of piezoelectric co-crystals. (iv) Co-crystals with high porosity demonstrate as expected low shear stiffness, and porous cocrystals also tend to host ordered clusters of confined polar solvent molecules. (v) Co-crystallization has consistently increased the dielectric constant of biomolecular crystals, which reduces the voltage output for energy harvesting applications but also increases the piezoelectric charge constants. Engineering co-crystals with larger dielectric constants could open up new avenues for these materials in conventional sensing and actuation applications currently dominated by ceramics. (vi) Racemic co-crystals to date have crystallized in centrosymmetric space groups that preclude piezoelectricity.

## Conclusions

In summary, we have explored the effect of chirality and co-assembly on the regulation of supramolecular packing of amino acid-based architectures, which resulted in tunable electromechanical properties including mechanical strength and piezoelectricity. Mirrored molecular packing was observed in the enantiopure assemblies showing similar physical properties, while racemic mixtures formed completely different packing modes determined by crystal structures. Moreover, co-assembly improved the thermal stability and mechanical strength due to the more tightly packed supramolecular organizations, where the highest values were found for the racemic co-assemblies of BPA/Ac-DL-Ala. Notably, racemic mixing could improve the mechanical strength of the assemblies compared with the enantiopure assemblies but did not result in piezoelectricity due to centrosymmetric space groups in the assemblies of Ac-DL-Ala and BPA/Ac-DL-Ala. Owing to the low symmetry and modest piezoelectric polarization, the enantiopure co-assemblies of BPA/Ac-L-Ala and BPA/Ac-D-Ala showed higher piezoelectric coefficients and power outputs in sandwich devices compared to the self-assemblies of Ac-L-Ala and Ac-D-Ala. This work demonstrates that both achiral centrosymmetric-crystallizing and right-handed D-amino acid molecules can be used to engineer highly piezoelectric crystalline assemblies. This expands the diverse molecular toolkit for the development of high-performance functional materials formed by bio-inspired minimalistic building blocks.

## Supporting Information Available

Detailed experimental procedures, optical microscopy images, PXRD patterns, TGA spectra, FTIR spectra, and ^1^H NMR spectra, (Figures S1–S16), and peak assignments, data collection and refinement statistics, and calculated piezoelectric charge tensor components (Tables S1–S8) (PDF)
