# Supplementary material for: Modulating the Electromechanical Response of Bio-Inspired Amino Acid-Based Architectures through Supramolecular Co-Assembly
Source: J Am Chem Soc. 2022 Sep 27;144(40):18375–86. doi: 10.1021/jacs.2c06321 (PMC9720716; doi:10.1021/jacs.2c06321)
Supplement: Supplementary file 1 — ja2c06321_si_001.pdf [file ja2c06321_si_001.pdf]

Supporting Information

Modulating the Electromechanical Response of Bio-Inspired Amino Acid-Based Architectures through Supramolecular Co-Assembly

Wei Ji,[*,†,#] Bin Xue,[§,#] Yuanyuan Yin,[◇,#] Sarah Guerin,[¶] Yuehui Wang,[†] Lei Zhang,[▽] Yuanqi Cheng,[§] Linda J. W. Shimon,[⊥] Yu Chen,[*,‡] Damien Thompson,[*,¶] Rusen Yang,[▽] Yi Cao,[§] Wei Wang,[§] Kaiyong Cai,[†] and Ehud Gazit[*,‡]

[†] Key Laboratory of Biorheological Science and Technology, Ministry of Education, College of Bioengineering, Chongqing University, Chongqing 400044, P. R. China.

[‡] The Shmunis School of Biomedicine and Cancer Research, George S. Wise Faculty of Life Sciences, Tel Aviv University, Tel Aviv 6997801, Israel.

[§] National Laboratory of Solid State Microstructure, Department of Physics, Nanjing University, Nanjing 210093, Jiangsu, China.

[◇] Chongqing Key Laboratory of Oral Diseases and Biomedical Sciences, Chongqing Municipal Key Laboratory of Oral Biomedical Engineering of Higher Education, Stomatological Hospital of Chongqing Medical University, Chongqing 401147, China.

[¶] Department of Physics, Bernal Institute, University of Limerick, V94 T9PX, Ireland.

[▽] CAEP Software Center for High Performance Numerical Simulation, Beijing 100088, China.

[⊥] Department of Chemical Research Support, Weizmann Institute of Science, Rehovot 76100, Israel.

[▽] School of Advanced Materials and Nanotechnology, Xidian University, Xi'an 710126, China.

Materials and Methods

Materials. All the solvents and chemicals are commercially available. Chemicals were used as received without any prior purification. Deionized water was purified by a Millipore water purification system (Darmstadt, Germany) with minimum resistivity of 18.2 MΩ cm. N-Acetyl-L-alanine (Ac-L-Ala), N-Acetyl-D-alanine (Ac-D-Ala), N-Acetyl-DL-alanine (Ac-DL-Ala), and 1,2-bis(4-pyridyl)ethane (BPA) were purchased from Sigma-Aldrich at a purity level of above 98%.

Crystal preparation. Crystals used for data collection were grown using slow solvent evaporation. BPA and Ac-Ala at 1: 2 molar ratio were first mixed and dissolved in methanol at a concentration of 20 mg/mL. Then, 2 mL was deposited in a glass vial and covered with Parafilm® in which 3 holes were pricked using a needle to permit solvent evaporation until crystal formation was observed at room temperature (~10 days).

Optical microscopy. The formed crystals (Ac-L-Ala, Ac-D-Ala, Ac-DL-Ala, BPA, BPA/Ac-L-Ala, BPA/Ac-D-Ala, BPA/Ac-DL-Ala) in a glass vial were viewed under a Nikon Ti-E inverted motorized microscope.

Powder X-ray diffraction (PXRD). PXRD spectra of the single and co-crystals (Ac-L-Ala, Ac-D-Ala, Ac-DL-Ala, BPA, BPA/Ac-L-Ala, BPA/Ac-D-Ala, BPA/Ac-DL-Ala) were analyzed using a BRUKER d8 ADVANCE DIFFRACTOMETER equipped with Goebels mirrors to parallelize the beam and a LYNXEYE-XE linear detector.

Thermogravimetric analysis (TGA). Thermal stability of all the single and co-crystal powder (Ac-L-Ala, Ac-D-Ala, Ac-DL-Ala, BPA, BPA/Ac-L-Ala, and BPA/Ac-D-Ala, BPA/Ac-DL-Ala) was evaluated by melting and decomposition temperatures using a Differential Scanning Calorimetry (NETZSCH STA 449F5, Germany) operating at a heating rate of 10 K min^{-1} in a flow of dry nitrogen at 10 mL min^{-1} over a range of 30 to 500 °C.

Circular dichroism (CD) spectroscopy. CD spectra of the single and co-crystal powder (Ac-L-Ala, Ac-D-Ala, Ac-DL-Ala, BPA, BPA/Ac-L-Ala, BPA/Ac-D-Ala, BPA/Ac-DL-Ala) were collected using an Applied Photophysics Chirascan Spectrometer with a bandwidth of 1.0 nm in the ultraviolet region of 190-400 nm using a 0.1 mm quartz cuvette. All samples were tested at a scanning speed of 40 nm min^{-1}

with a data spacing of 0.5 nm at ambient temperature. CD spectra were obtained by subtracting the blank background.

Fourier-transform infrared (FTIR) spectroscopy. The single and co-crystals (Ac-L-Ala, Ac-D-Ala, Ac-DL-Ala, BPA, BPA/Ac-L-Ala, BPA/Ac-D-Ala, BPA/Ac-DL-Ala) were deposited onto a disposable crystal KBr IR card, (International Crystal Labs, Garfield, New Jersey, USA). The FTIR spectra were collected using a Nicolet iS50 FTIR spectrometer (Thermo Scientific, Waltham, Massachusetts, USA) from 4000 to 400 cm^{-1}. The background signal was recorded without a sample and subtracted to obtain each FTIR spectrum.

Nuclear magnetic resonance (NMR). The powder of single and co-crystal (Ac-L-Ala, Ac-D-Ala, Ac-DL-Ala, BPA, BPA/Ac-L-Ala, BPA/Ac-D-Ala, BPA/Ac-DL-Ala) was dissolved in D_2O at a concentration of 5mg/mL. ^1H NMR spectra were recorded using Bruker Advance NEO 400 MHz spectrometers at 278 K. ^1H NMR chemical shifts are reported relative to Me_4Si and were referenced via residual proton resonances of the corresponding deuterated solvent (D_2O). Chemical shifts are measured in ppm and the spectra are calibrated using the residual D_2O signals.

Single crystal X-ray diffraction. Crystals were coated in Paratone oil (Hampton Research), mounted on a MiTeGen cryo-loop, and flash-frozen in liquid nitrogen. Diffraction data were collected at 100 K on a Bruker KappaApexII system using Mo Kα radiation, λ = 0.710 73 Å.

Processing and structural refinement of crystal data. The diffraction data were analyzed using the Bruker Apex2 suite. The structure was solved by direct methods using SHELXT-2014/5.59. The refinements were measured with SHELXL-2016/4 and weighted full-matrix least-squares against $|F^2|$ using all data. Atoms were refined independently and anisotropically, with the exception of hydrogen atoms, which were placed in calculated positions and refined in riding mode. Crystal data collection and refinement parameters are shown in Table S1 and the complete data can be found in the cif file as supplementary information.

Atomic force microscopy (AFM) nano-indentation experiments. All the AFM nanoindentation experiments were performed using a commercial AFM (JPK,

Nanowizard IV, Berlin, Germany). QI mode (conditions: pixels: 126 × 126; Z length: 0.3 μm; extend and retract speed: 30 μm s^{-1}; Z resolution: 80000 Hz; maximum loading force: 800 nN) and RTESPA-525 cantilevers (Bruker Company, half-open angle of the pyramidal face of θ: < 10°, tip radius: ~10 nm, spring constant: ~200 N m^{-1}) were used in the experiments. Typically, the samples were fixed on a mica substrate and the cantilever was moved above the crystals with the help of a microscope. Then, the cantilever approached the surface of the crystal and retracted, and the force-displacement curves were recorded during the process. The indentation depths pressed by the cantilever tip were less than 10 nm for all the samples. Young's modulus of crystals could be calculated by fitting the extended curve with the Hertz model (1).

$$F = \frac{4}{3}\frac{E}{(1-v^2)}\sqrt{R}\delta^{3/2} \quad (1)$$

In which F corresponds to the force, δ corresponds to the depth of the crystal pressed by the cantilever tip, R corresponds to the radius of the tip, E is the Young's modulus of the crystals and ν is the Poisson ratio (ν = 0.3). The point stiffness was calculated from the force-displacement curves after subtracting the deformation of the cantilever. For each sample, more than 6 regions were randomly selected to perform the experiments. Tip to tip dependency was excluded by using more than two cantilevers in each experiments. All the data was analyzed and the two-dimensional diagrams were reconstructed using the commercial software (JPK data processing 7.0.46) provided by JPK company.

Point stiffness measurement. The cantilever and crystal in the AFM experiments could be considered as two serial springs. Thus, the point stiffness was directly calculated from the extend curves (k_{means}), the stiffness of cantilever (k_{can}) and the crystal stiffness (k_{cry}) following equation (2). After the calculation of crystal stiffness using equation (2), the stiffness histograms were also constructed.

$$k_{cry} = \frac{k_{can} \cdot k_{means}}{k_{can} - k_{means}} \quad (2)$$

Piezoelectricity prediction. Electromechanical properties were predicted from periodic density functional theory (DFT) calculations[1] on the range of BPA/AcA chiral

co-crystals and their unimolecular component crystals using the VASP code[2]. Electronic structures were calculated using the PBE functional[3] with Grimme-D3 dispersion corrections[4] and projector augmented wave (PAW) pseudopotentials[5]. The crystal structures were optimized using a plane wave cut-off of 600 eV with a 4x4x4 k-point grid. A finite differences method was used to calculate the stiffness tensor, with each atom being displaced in each direction by ± 0.01 Å, and piezoelectric strain constants and dielectric tensors were calculated using Density Functional Perturbation Theory[6] (DFPT), with a plane wave cut-off of 600 eV and k-point sampling of 2x2x2. Young's moduli were derived from the stiffness tensor and its inverse compliance matrix components. Values are presented as a Voigt-Reuss-Hill average[7, 8]. Crystal structures were visualized using VESTA[9].

Fabrication and power generation of a piezoelectric generator. Crystal powders were filled into a PDMS (Sylgard®184) protective layer with a filling area of 1.2x1.2 cm^2, which was sandwiched between two copper attached silicon substrates serving as top and bottom electrodes, respectively. A PDMS film was coated on the smooth surface of Kapton tape. A strip of double-sided tape was attached to the PDMS film. Another silicon substrate with the copper electrode was closely adsorbed on the double-sided tape to complete the device structure. No gap was left in the device. Copper wires were connected to the electrodes of the device and PDMS was coasted on the electrodes as buffer. For power generation test, the power generator filled with crystals was firmly fixed onto a stainless-steel plate. Cyclic forces were periodically applied on the device with force control. Al foil was used to avoid triboelectric signals. The electrical signal from the devices was collected using an electrometer (Keithley 6514) and a data acquisition system (NI USB-6218).

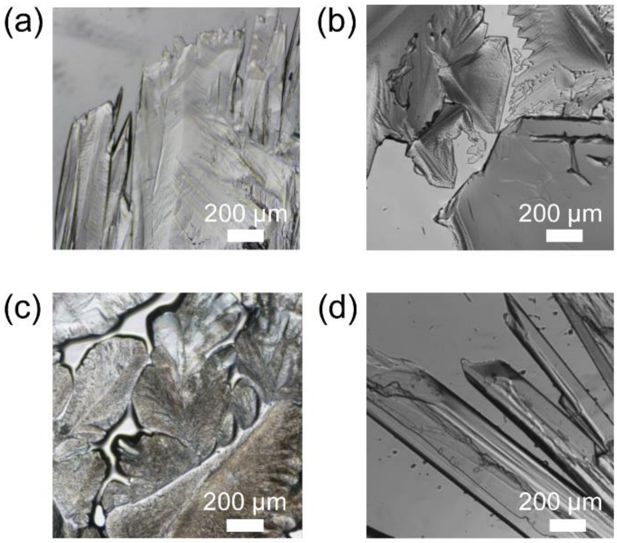

Figure S1. Optical microscopy images of (a) Ac-D-Ala, (b) Ac-L-Ala, (c) Ac-DL-Ala and (d) BPA/Ac-L-Ala crystals.

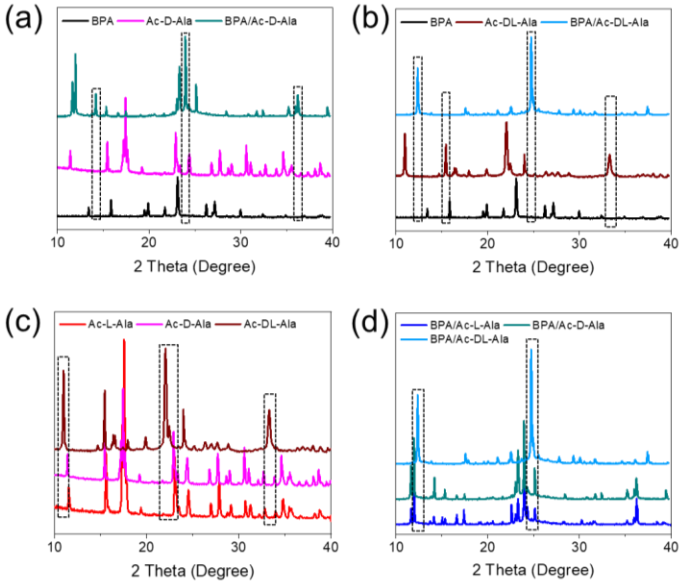

Figure S2. PXRD patterns for the self- and co-assemblies.[12] (a) BPA, Ac-D-Ala, BPA/Ac-D-Ala. (b) BPA, Ac-DL-Ala, BPA/Ac-DL-Ala. (c) Ac-L-Ala, Ac-D-Ala, Ac-DL-Ala. (d) BPA/Ac-L-Ala, BPA/Ac-D-Ala, BPA/Ac-DL-Ala.

Table S1. Peak assignments (measured in detector angle of 2 theta) of powder XRD patterns.

BPA	L-AcA	BPA/L-AcA	D-AcA	BPA/D-AcA	DL-AcA	BPA/DL-AcA
2θ (º)	2θ (º)	2θ (º)	2θ (º)	2θ (º)	2θ (º)	2θ (º)
13.40	11.52	11.66	11.40	11.66	10.96	12.36
15.83	15.55	11.97	15.44	11.96	15.44	17.58
19.49	17.32	14.13	17.21	14.19	16.37	21.07
19.88	17.54	15.03	17.43	15.33	16.57	22.55
21.71	19.37	16.63	19.23	17.47	17.95	24.72
23.10	23.04	17.40	22.90	23.01	19.89	27.78
26.23	24.50	22.57	24.40	23.30	22.03	29.34
27.15	26.97	23.30	26.82	23.95	22.46	30.06
29.94	27.87	23.96	27.72	25.10	23.98	31.71
32.37	29.14	24.31	29.02	28.44	26.36	37.43
	30.70	25.14	30.58	31.73	26.66	
	31.24	36.14	31.07	32.40	33.30	
	32.80		32.66	35.20		
	34.77		34.60	36.20		
			35.52	39.42		
			38.64			

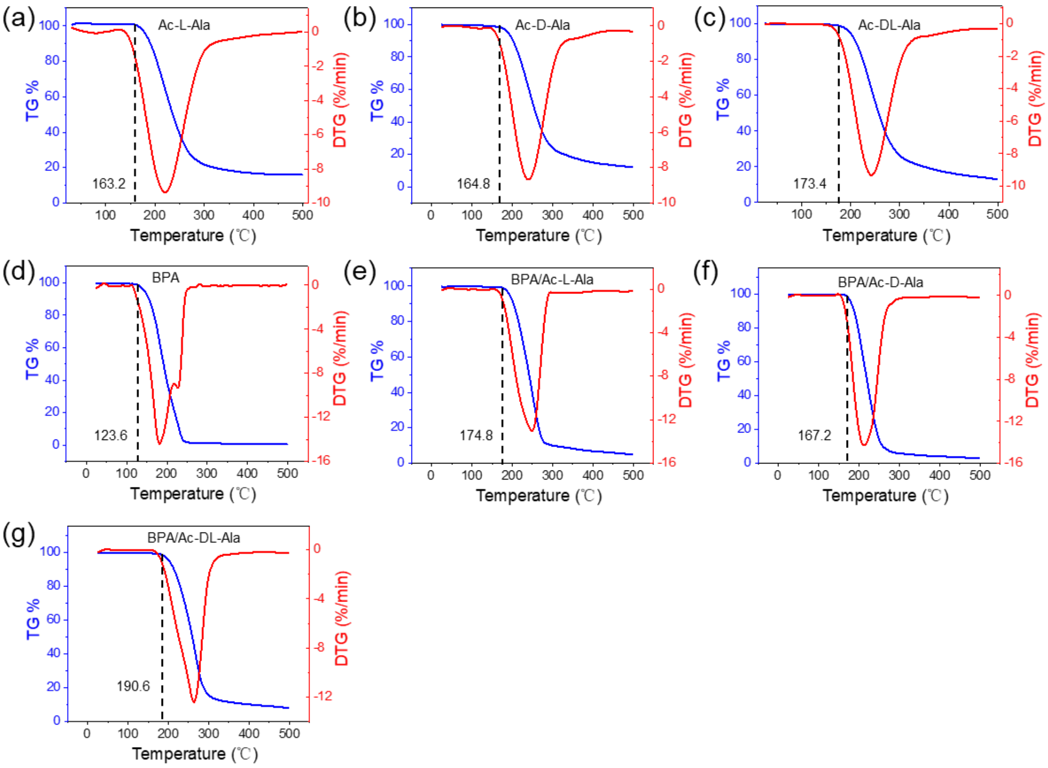

Figure S3. TGA spectra of all the crystals. (a) Ac-L-Ala, (b) Ac-D-Ala, (c) Ac-DL-Ala, (d) BPA, (e) BPA/Ac-L-Ala, (f) BPA/Ac-D-Ala, (g) BPA/Ac-DL-Ala.

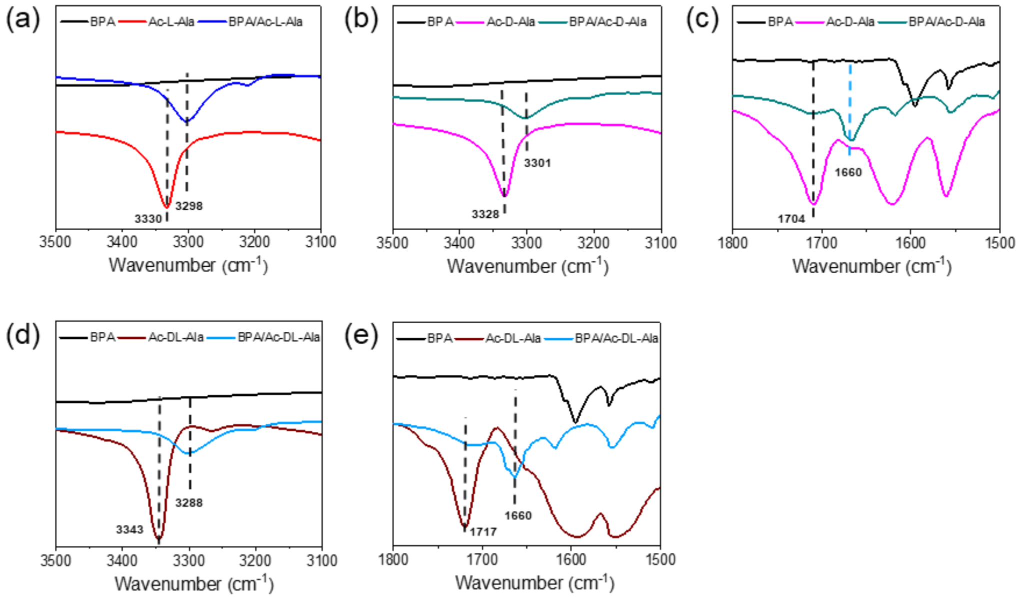

Figure S4. FTIR spectra of single and co-crystals.[12] (a) BPA, Ac-L-Ala, BPA/Ac-L-Ala. (b, c) BPA, Ac-D-Ala, BPA/Ac-D-Ala. (d, e) BPA, Ac-DL-Ala, BPA/Ac-DL-Ala.

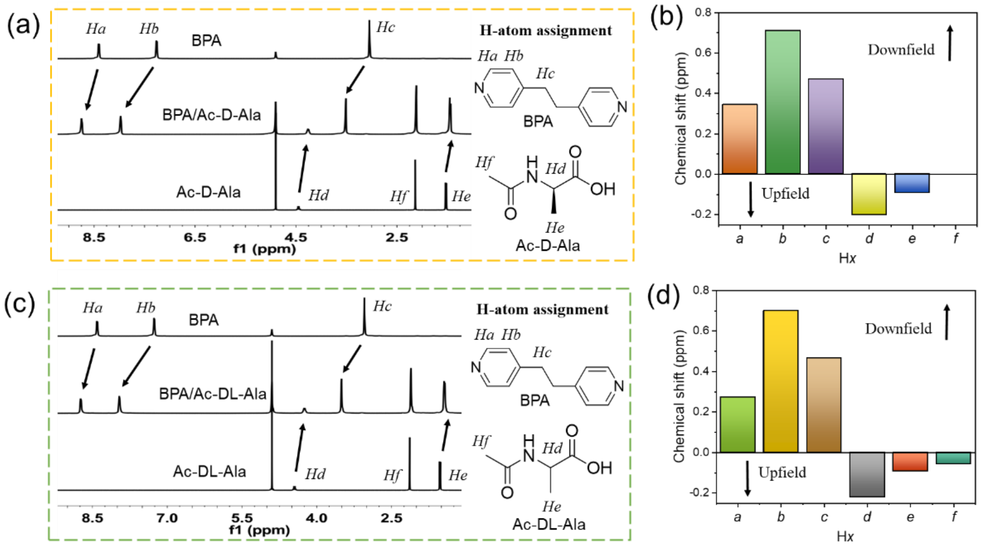

Figure S5. (a, c) ^{1}H NMR spectra of (a) BPA, BPA/Ac-D-Ala, and Ac-D-Ala, (c) BPA, BPA/Ac-DL-Ala, and Ac-DL-Ala. (b, d) Chemical shift of ^{1}H NMR of (b) Ac-D-Ala, and (d) Ac-DL-Ala compared to the single components. [12]

Table S2. Data collection and refinement statistics for X-ray crystallography of the co-crystals.

Crystal data	BPA/Ac-D-Ala	BPA/Ac-DL-Ala
Sum formula	C22 H32 N4 O7	C44 H60 N8 O12
Mr	464.51	893.00
Crystal system	Orthorhombic	Triclinic
Space group	*C*222	*P* -1
a (Å)	13.9215(5)	11.5465(7)
b (Å)	14.3075(6)	13.9304(8)
c (Å)	11.7595(5)	14.9658(9)
α (°)	90	90.915(2)
β (°)	90	109.332(2)
γ (°)	90	90.144(2)

Z	4	2
Mυ (mm^{-1})	0.099	0.096
F000	992.0	952.0
Temperature (K)	100	100
Wavelength λ= (Å)	0.71073	0.71073
*T*min , *T*max	0.977,0.980	0.986,0.992
θ_{max} (°)	30.636	25.712
No. of reflections collected(unique)	9615(3601)	24047(8595)
Rint	0.0199	0.0431
Data/restraints/parameters	3601/0/155	8595/0/589
Completeness to θ (%)	99.3	99.6
Goodness-of-fit on F^2	1.073	1.055
Final R_1 and wR_2 indices [I>2σ(I)]	0.0382 0.1082	0.0670 0.1604
R_1 and wR_2 indices (all data)	0.0409 0.1105	0.1071 0.1787
No. CCDC	2127127	2150462

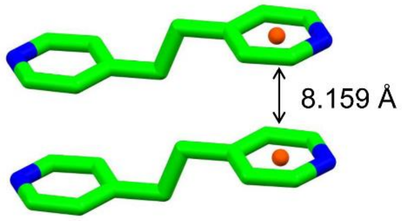

Figure S6. Centroid-to-centroid distance between aromatic rings in the BPA crystal structure (CCDC number: 125942).[10]

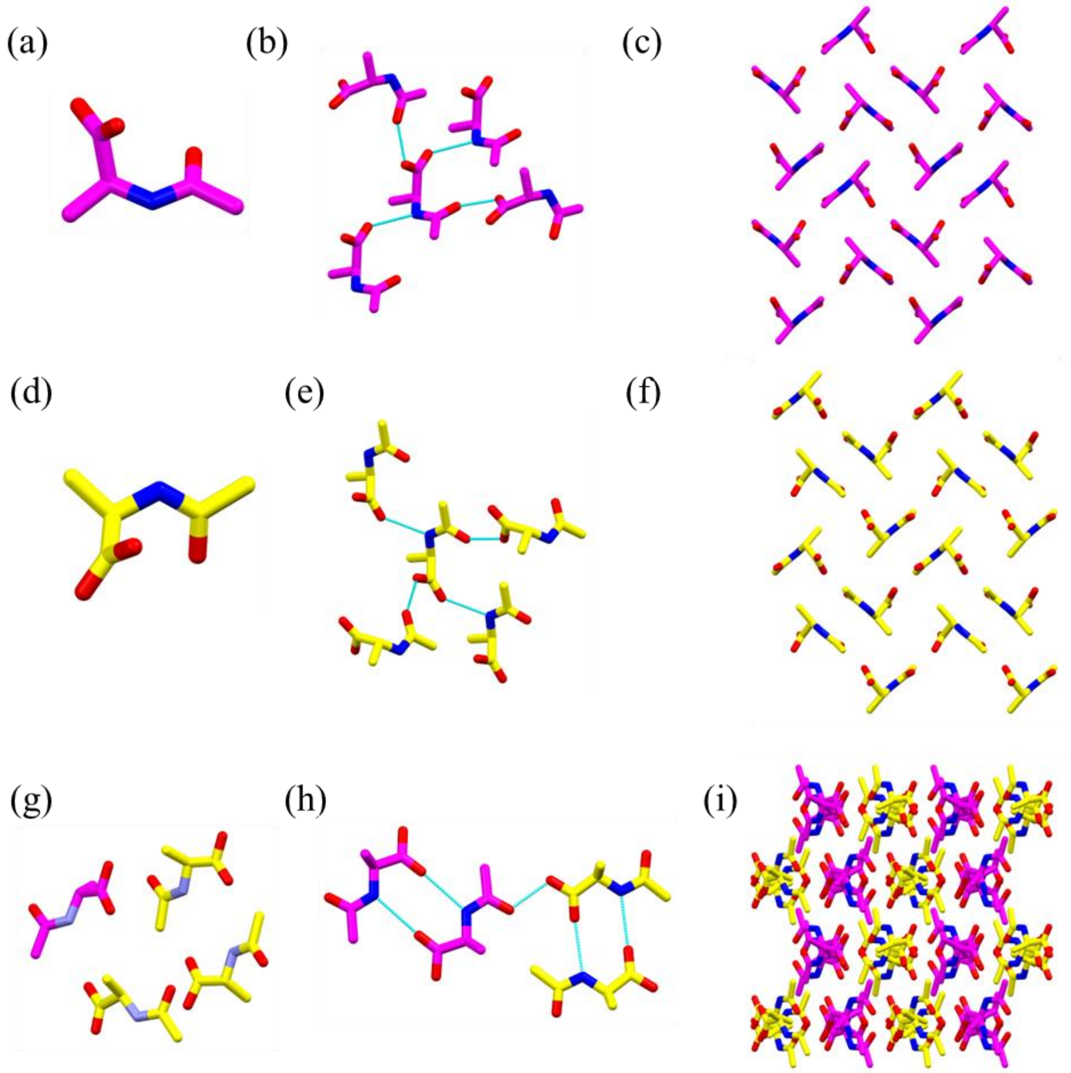

Figure S7. (a-c) Crystal structure of Ac-L-Ala: (a) Asymmetric unit, (b) Intermolecular hydrogen bonding formation between Ac-L-Ala molecules, (c) Higher-order molecular packing in the crystallographic *c* direction. (d-f) Crystal structure of Ac-D-Ala: (d) Asymmetric unit, (e) Intermolecular hydrogen bonding formation between Ac-D-Ala molecules, (f) Higher-order molecular packing in the crystallographic *c* direction. (g-i) Crystal structure of Ac-L-Ala: (g) Asymmetric unit, (h) Intermolecular hydrogen bonding formation between Ac-L-Ala molecules, (i) Higher-order molecular packing in the crystallographic *c* direction. The CCDC numbers are 239282, 1994775, 1994776 for Ac-L-Ala, Ac-D-Ala, Ac-DL-Ala, respectively.[11-12]

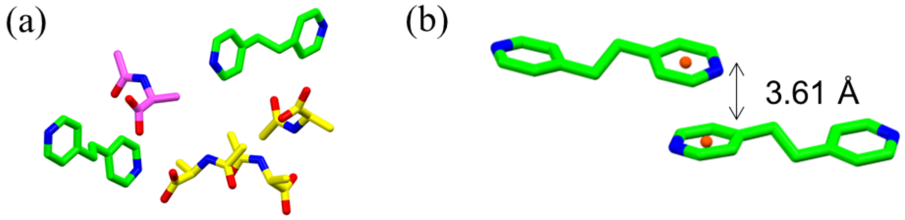

Figure S8. (a) Asymmetric unit of BPA/Ac-DL-Ala co-crystal. (b) Centroid-to-centroid distance between aromatic rings (J-aggregation) in the BPA/Ac-DL-Ala co-crystal.

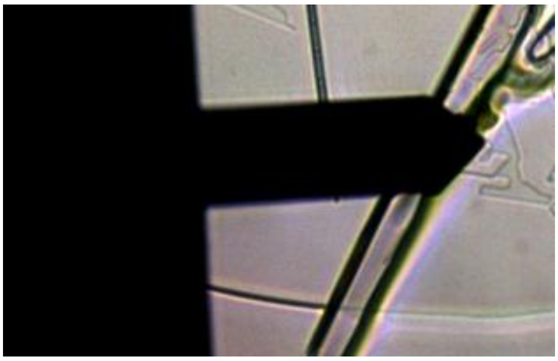

Figure S9. AFM indentation experiments: crystals were mounted on the surface of the substrates and the cantilever was extended to the surface of the crystal with the help of an optical microscope.

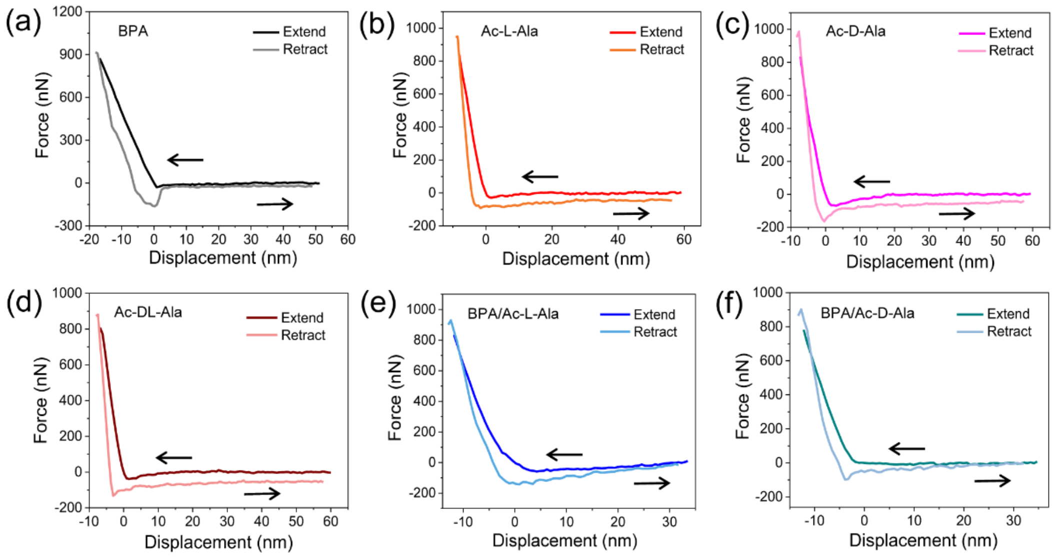

Figure S10. Typical force-displacement traces of (a) BPA, (b) Ac-L-Ala, (c) Ac-D-Ala, (d) Ac-DL-Ala, (e) BPA/Ac-L-Ala, and (f) BPA/Ac-D-Ala after subtracting the deformation of the cantilever. The left arrow represents expansion and the right arrow represents retraction.

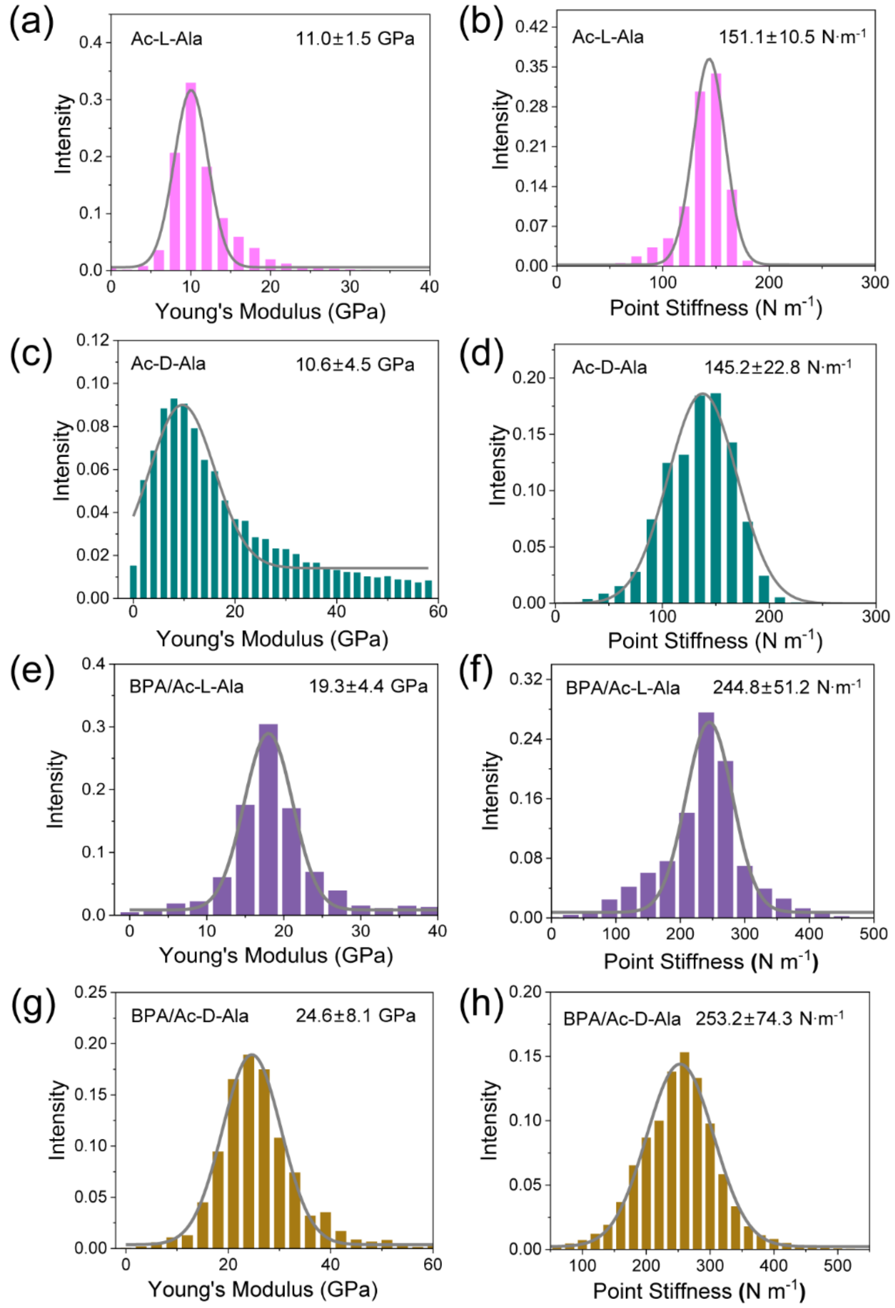

Figure S11. (a, c, e, g) Statistical Young's moduli distributions. (a) Ac-L-Ala. (c) Ac-D-Ala. (e) BPA/Ac-L-Ala. (g) BPA/Ac-D-Ala. (b, d, f, h) Statistical point stiffness distributions. (b) Ac-L-Ala. (d) Ac-D-Ala. (f) BPA/Ac-L-Ala. (h) BPA/Ac-D-Ala.

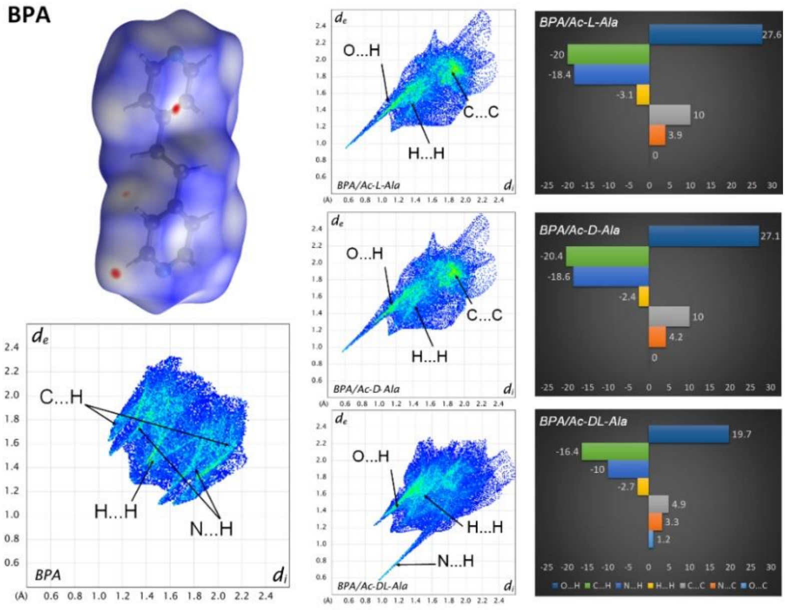

Figure S12. Hirshfeld surface, fingerprint plot, and individual atomic contact percentage contribution to the Hirshfeld surface for the BPA molecule in BPA/Ac-L-Ala, BPA/Ac-D-Ala, and BPA/Ac-DL-Ala co-crystals. The atomic contact percentage contribution to the Hirshfeld surface in the pristine BPA crystal was set as a reference.

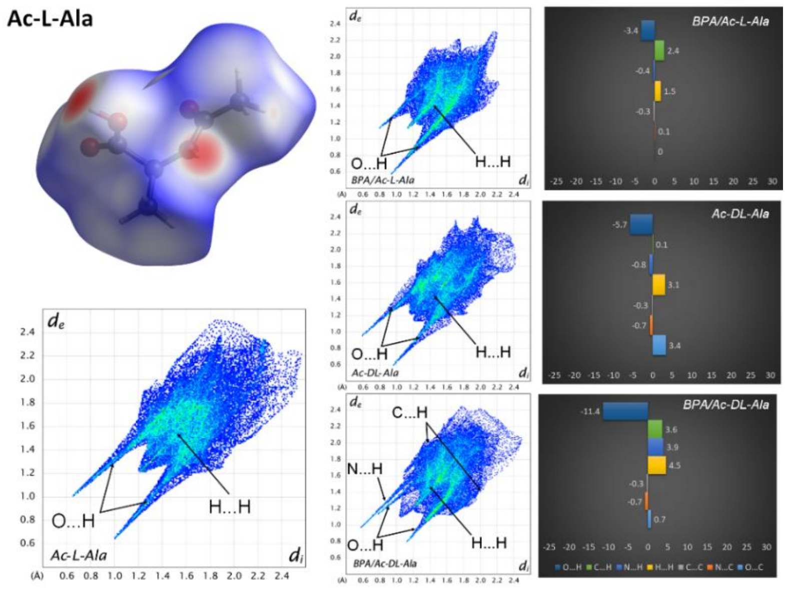

Figure S13. Hirshfeld surface, fingerprint plot, and individual atomic contact percentage contribution to the Hirshfeld surface for the Ac-L-Ala molecule in BPA/Ac-L-Ala, Ac-DL-Ala, and BPA/Ac-DL-Ala co-crystals. The atomic contact percentage contribution to the Hirshfeld surface in the pristine Ac-L-Ala crystal was set as a reference.

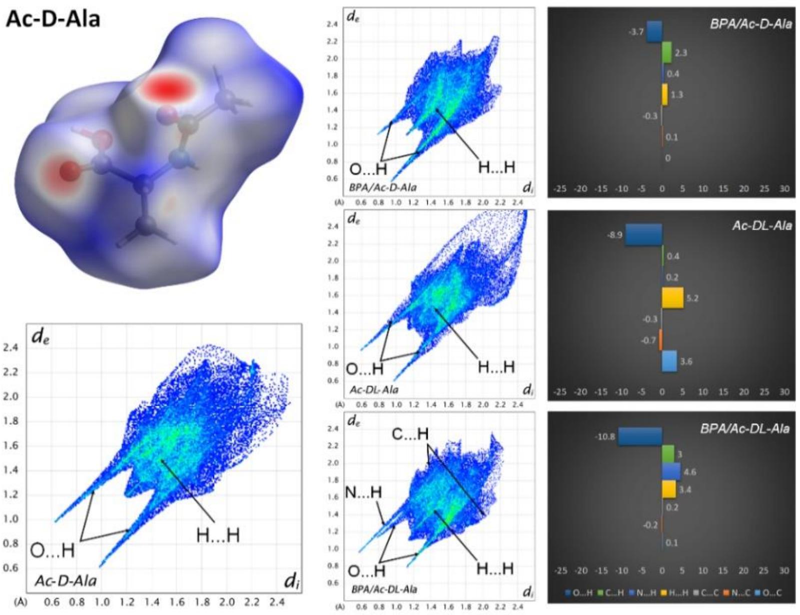

Figure S14. Hirshfeld surface, fingerprint plot, and individual atomic contact percentage contribution to the Hirshfeld surface for the Ac-D-Ala molecule in BPA/Ac-D-Ala, Ac-DL-Ala, and BPA/Ac-DL-Ala co-crystals. The atomic contact percentage contribution to the Hirshfeld surface in the pristine Ac-D-Ala crystal was set as a reference.

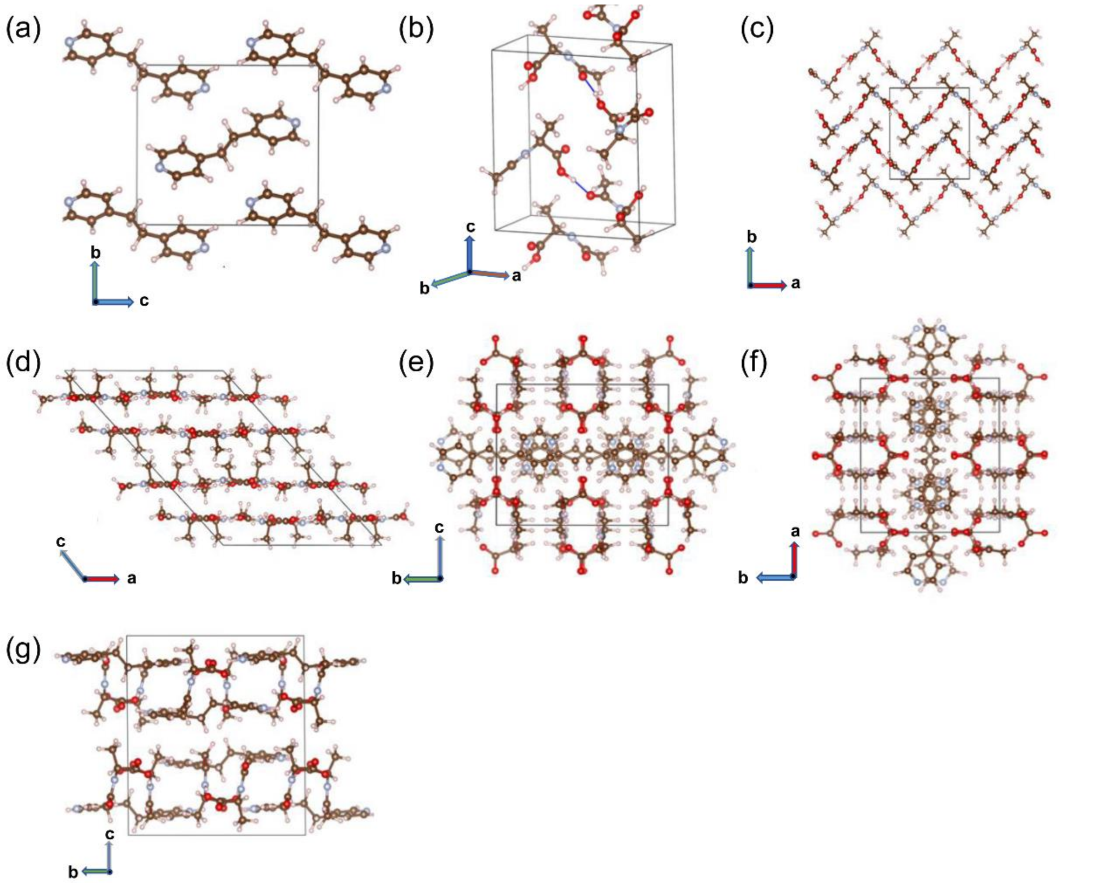

Figure S15. DFT-calculated supramolecular packing modes. Molecules are shown in CPK representation, with hydrogen in white, oxygen in red, carbon in brown, and nitrogen in blue. (a) BPA unit cell. (b) Ac-L-Ala unit cell. (c) Ac-D-Ala unit cell. (d) Ac-DL-Ala unit cell. (e) BPA/Ac-L-Ala unit cell. (f) BPA/Ac-D-Ala unit cell. (g) BPA/Ac-DL-Ala unit cell.

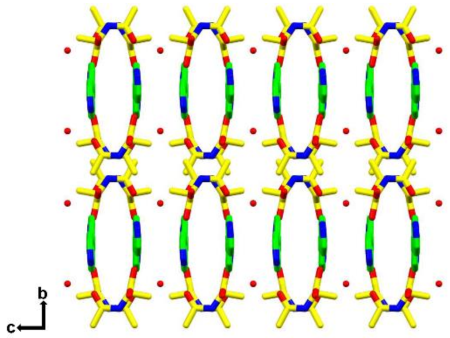

Figure S16. The porous structure of BPA/Ac-D-Ala co-crystals.

Table S3. Calculated piezoelectric charge tensor components e_{ij} (in units of C/m^2), strain tensor components d_{ik} (pm/V), and voltage tensor components g_{ij} (mV m/N), of Ac-L-Ala single crystal.

**Charge Tensor (C/m^2)**					
0.00	0.00	0.00	-0.004	0.00	0.00
0.00	0.00	0.00	0.00	-0.01	0.00
0.00	0.00	0.00	0.00	0.00	0.04
**Strain Tensor (pm/V)**					
0.00	0.00	0.00	-1.30	0.00	0.00
0.00	0.00	0.00	0.00	-2.10	0.00
0.00	0.00	0.00	0.00	0.00	2.90
**Voltage Tensor (mV m/N)**					
0.00	0.00	0.00	-39	0.00	0.00
0.00	0.00	0.00	0.00	-67	0.00
0.00	0.00	0.00	0.00	0.00	109

Table S4. Calculated piezoelectric charge tensor components e_{ij} (in units of C/m^2), strain tensor components d_{ik} (pm/V), and voltage tensor components g_{ij} (mV m/N), of Ac-D-Ala single crystal.

**Charge Tensor (C/m^2)**					
0.00	0.00	0.00	0.02	0.00	0.00
0.00	0.00	0.00	0.00	0.01	0.00
0.00	0.00	0.00	0.00	0.00	-0.02
**Strain Tensor (pm/V)**					
0.00	0.00	0.00	5.30	0.00	0.00
0.00	0.00	0.00	0.00	1.20	0.00
0.00	0.00	0.00	0.00	0.00	-1.30
**Voltage Tensor (mV m/N)**					
0.00	0.00	0.00	163	0.00	0.00
0.00	0.00	0.00	0.00	40	0.00
0.00	0.00	0.00	0.00	0.00	-50

Table S5. Calculated piezoelectric charge tensor components e_{ij} (in units of C/m^2), strain tensor components d_{ik} (pm/V), and voltage tensor components g_{ij} (mV m/N), of the BPA/Ac-L-Ala cocrystal.

**Charge Tensor (C/m^2)**					
0.00	0.00	0.00	0.03	0.00	0.00
0.00	0.00	0.00	0.00	0.05	0.00
0.00	0.00	0.00	0.00	0.00	0.01
**Strain Tensor (pm/V)**					
0.00	0.00	0.00	21.9	0.00	0.00
0.00	0.00	0.00	0.00	10.3	0.00
0.00	0.00	0.00	0.00	0.00	4.80
**Voltage Tensor (mV m/N)**					
0.00	0.00	0.00	477	0.00	0.00
0.00	0.00	0.00	0.00	283	0.00
0.00	0.00	0.00	0.00	0.00	129

Table S6. Calculated piezoelectric charge tensor components e_{ij} (in units C/m^2), strain tensor components d_{ik} (pm/V), and voltage tensor components g_{ij} (mV m/N), of the BPA/Ac-D-Ala cocrystal.

**Charge Tensor (C/m^2)**					
0.00	0.00	0.00	-0.03	0.00	0.00
0.00	0.00	0.00	0.00	-0.08	0.00
0.00	0.00	0.00	0.00	0.00	0.01
**Strain Tensor (pm/V)**					
0.00	0.00	0.00	-26.3	0.00	0.00
0.00	0.00	0.00	0.00	-14.9	0.00
0.00	0.00	0.00	0.00	0.00	4.90
**Voltage Tensor (mV m/N)**					
0.00	0.00	0.00	-545	0.00	0.00
0.00	0.00	0.00	0.00	-363	0.00
0.00	0.00	0.00	0.00	0.00	137

Table S7. The magnitude of the predicted piezoelectric strain constants of BPA, Ac-L-Ala, Ac-D-Ala, Ac-DL-Ala, BPA/Ac-L-Ala, BPA/Ac-D-Ala, and BPA/Ac-DL-Ala.

**Sample**	**d_{14}**	**d_{15}**	**d_{24}**	**d_{25}**	**d_{31}**	**d_{32}**	**d_{33}**	**d_{36}**
BPA	0	0	0	0	0	0	0	0
Ac-L-Ala	1.3	0	0	2.1	0	0	0	2.9
Ac-D-Ala	5.3	0	0	1.2	0	0	0	1.3
Ac-DL-Ala	0	0	0	0	0	0	0	0
BPA/Ac-L-Ala	21.9	0	0	10.3	0	0	0	4.8
BPA/Ac-D-Ala	26.3	0	0	14.9	0	0	0	4.9
BPA/Ac-DL-Ala	0	0	0	0	0	0	0	0

Table S8. The piezoelectric coefficient d_{14} of different biological and non-biological materials, as well as the BPA/Ac-L-Ala, BPA/Ac-D-Ala crystals.

Material type	Material name	Piezoelectric coefficient of d_{14} (pC/N)	Reference
Organic crystals	BPA/Ac-L-Ala	21.9	This work
	BPA/Ac-D-Ala	26.3	This work
Biological materials	L-alanine	6	[13]
	Collagen	12	[14]
	Hydroxyapatite	14	[15]
Non-biological materials	Poly-lactic acid	11	[16]
	$Ca_3TaGa_3Si_2O_{14}$	14	[17]
	$CdTeMoO_6$	20	[18]

References

[1] Argaman, N.; Makov, G. Density functional theory: An introduction. *Am. J. Phys.* **2000**, *68*, 69-79.

[2] Hafner, J. Materials simulations using VASP-a quantum perspective to materials science. *Comput. Phys. Commun.* **2007**, *177*, 6-13.

[3] Perdew, J. P.; Chevary, J.; Vosko, S.; Jackson, K. A.; Pederson, M. R.; Singh, D.; Fiolhais, C. Atoms, molecules, solids, and surfaces: Applications of the generalized gradient approximation for exchange and correlation. *Phys. Rev. B.* **1992**, *46*, 6671.

[4] Grimme, S.; Antony, J.; Ehrlich, S.; Krieg, H. A consistent and accurate ab initio parametrization of density functional dispersion correction (DFT-D) for the 94 elements H-Pu. *J. Chem. Phys.* **2010**, *132*, 154104.

[5] Kresse, G.; Joubert, D. From ultrasoft pseudopotentials to the projector augmented-wave method. *Phys. Rev. B.* **1999**, *59*, 1758.

[6] Wu, X.; Vanderbilt, D.; Hamann, D. Systematic treatment of displacements, strains, and electric fields in density-functional perturbation theory. *Phys. Rev. B.* **2005**, *72*, 035105.

[7] Chung, D.; Buessem, W. The voigt-reuss-hill approximation and elastic moduli of polycrystalline MgO, CaF_2, β-ZnS, ZnSe, and CdTe. *J. Appl. Phys.* **1967**, *38*, 2535-2540.

[8] Zuo, L.; Humbert, M.; Esling, C. Elastic properties of polycrystals in the Voigt-Reuss-Hill approximation. *J. Appl. Crystallogr.* **1992**, *25*, 751-755.

[9] Momma, K.; Izumi, F. J. VESTA 3 for three-dimensional visualization of crystal, volumetric and morphology data. *J. Appl. Crystallogr.* **2011**, *44*, 1272-1276.

[10] Ide, S.; Karacan, N.; Tufan, Y. 1, 2-Bis (4-pyridyl) ethane. *Acta Crystallogr., Sect. C:* **1995**, *51*, 2304-2305.

[11] Gorbitz, C. H.; Sagstuen, E. N-acetyl-L-alanine. *Acta Crystallogr., Sect. E: Struct. Rep. Online*, **2004**, *60*, o860-o862.

[12] Ji, W.; Xue, B.; Bera, S.; Guerin, S.; Shimon, L.J. W.; Ma, Q.; Tofail, S. A. M.; Thompson, D.; Cao, Y.; Wang, W.; Gazit, E. Modulation of physical properties of organic cocrystals by amino acid chirality. *Mater. Today* **2021**, *42*, 29-40.

[13] Guerin, S. S.; Tofail, A.; Thompson, D. Longitudinal piezoelectricity in orthorhombic amino acid crystal films. *Cryst. Growth Des.* **2018**, *18*, 4844-4848.

[14] Denning, D.; Kilpatrick, J. I.; Fukada, E.; Zhang, N.; Habelitz, S.; Fertala, A.; Rodriguez, B. Piezoelectric tensor of collagen fibrils determined at the nanoscale. *ACS Biomater. Sci. Eng.* **2017**, *3*, 929-935.

[15] García1, E. I.; Miguel, C. T. S.; Merino, J. G. Mechano-optical analysis of piezoelectric hydroxyapatite composites. *J. Phys.: Conf. Ser.* **2021**, 1723, 012009.

[16] Farahani, A.; Zarei-Hanzaki, A.; Abedi, H.R.; Haririan, I.; Akrami, M.; Aalipour, Z.; Tayebi, L. An investigation into the polylactic acid texturization through thermomechanical processing and the improved d_{33} piezoelectric outcome of the fabricated scaffolds. *J. Mater. Res. Technol.* **2021**, *15*, 6356-6366.

[17] Xiong, K. N.; Wang, S.; Tu, X. N.; Man, Z. Y.; Zheng, Y. Q.; Karaki, T.; Shia, E. W. Growth and piezoelectric properties of large sized $Ca_3TaGa_3Si_2O_{14}$ crystals. *CrystEngComm*, **2021**, *23*, 5362-5366.

[18] Li, C. G.; Gao, Z. L.; Tian, X. X.; Wu, Q.; Lu, W. Q.; Sun, Y. X.; Tao, X. T. Electric-elastic properties of a novel high-quality $CdTeMoO_6$ piezoelectric crystal. *CrystEngComm* **2018**, *20*, 5602-5608.
